# Japanese encephalitis virus-associated human microglia induce cell death of human microvascular endothelial cells in receptor-independent infection

**DOI:** 10.3389/fcimb.2025.1580958

**Published:** 2025-05-02

**Authors:** Isabelle Fellay, Pauline Blanc, Alexey Larionov, Léa Schlunke, Luis Filgueira, Nils Lannes

**Affiliations:** ^1^ Department of Oncology, Microbiology and Immunology, Unit of Anatomy, Faculty of Science and Medicine, University of Fribourg, Fribourg, Switzerland; ^2^ Laboratory of Microbiology and Microtechnology, School of Life Sciences (SV), Ecole Polytechnique Fédérale de Lausanne, Lausanne, Switzerland

**Keywords:** Japanese encephalitis (JE) virus, human microglia, microvascular endothelial cells, receptor-independent infection, intercellular interactions, cytotoxicity, TRAIL (TNF-related apoptosis-inducing ligand)

## Abstract

**Introduction:**

The neurotropic virus Japanese encephalitis virus invades the human central nervous system, inducing neuroinflammation and further disruption of the blood-brain barrier. JEV interacts with various cell types of the blood-brain barrier including the endothelial cells. The present work aims to investigate impact of receptor-dependent and independent infection of human microvascular endothelial cells by Japanese encephalitis virus.

**Methods:**

Receptor-dependent infection was achieved using cell-free virus while receptor-independent infection was by co-culture of microvascular endothelial cells with virus-associated microglia.

**Results:**

While both receptor-dependent and independent infections of human microvascular endothelial cells led to virus propagation, only receptor-independent infection induced cell death of human microvascular endothelial cells. While the CX_3_CR1-CX_3_CL1 axis was inefficient in blocking virus rescue and protecting endothelial cell from cell death, transcriptomics analysis identified Tumour Necrosis Factor-related apoptosis inducing ligand and receptors as potential key player leading to endothelial cell death.

**Discussion:**

Overall, our findings demonstrate that human microvascular endothelial cells supply virus propagation and Japanese encephalitis virus-associated microglia greatly contribute to endothelial cell death, an important component of the blood brain barrier integrity. Importantly, Tumour Necrosis Factor-related apoptosis inducing ligand and receptors represents a promising therapeutic target preventing microvascular endothelial cell death after neuroinvasion.

## Introduction

Japanese encephalitis virus (JEV) is a neurotropic flavivirus responsible for Japanese encephalitis (JE), an uncontrolled inflammatory disease of the central nervous system (CNS). JEV is transmitted by mosquito vectors in a zoonotic life cycle including pig amplifiers and water bird reservoir hosts (Solomon, 2004). Humans are accidental dead-end hosts due to low viremia undermining further virus transmission (Misra and Kalita, 2010). According to current WHO website numbers, JE annual incidence is of 68,000 cases with a 20-35% mortality rate and 20-30% of survivors suffering life-treating neurological problems. JEV is endemic in northern regions and epidemic in southern regions of the Asia-Pacific, counting most of JE cases (Wang and Liang, 2015). Despite the availability of efficient vaccines, resurgence of JE cases occurred in Australia in 2022 (Srivastava et al., 2023). In future years, JEV may become a worldwide public health concern due to spreading of competent mosquito vectors, detection of JEV in other geographical regions and potential vector-free transmission and persistence of JEV in pigs (Monath, 2023; Gossner et al., 2024; Ricklin et al., 2016).

JEV interacts with various cells types in a receptor-dependent manner necessitating the binding of the envelop (E) protein of JEV to its still unidentified receptor on a target cell (Yun and Lee, 2018). For instance, endothelial cells of the blood-brain barrier (BBB) are susceptible to JEV (Shwetank et al., 2013). Within the CNS, JEV preferentially targets developing neuronal cells (Kimura-Kuroda et al., 1993). Besides, JEV interacts with microglia (Thongtan et al., 2012), a unique CNS resident immune cell type (Ginhoux et al., 2013; Lannes et al., 2017a), but infection is abortive (Lannes et al., 2017b). Prominently, JEV-infected human microglia can transmit infectious JEV material to susceptible target cells in a receptor-independent infection manner leading to the rescue of infectious viral particles (Lannes et al., 2017b; Lannes et al., 2019). At the BBB, microglia naturally interact with various cell types including endothelial cells (Sheikh et al., 2019), a potential candidate target cell type in intercellular virus transmission. Upon JEV pathogenesis, neuroinvasion precedes neuroinflammation and breakdown of the BBB (Li et al., 2015). Therefore, the disruption of the BBB may result from the interaction between infected cells of the CNS and of the BBB such as microglia and endothelial cells, respectively.

The present study aims to investigate the impact of a receptor-dependent and a receptor-independent JEV infection of human microvascular endothelial cells on virus propagation and cell death. In order to achieve this work, direct infection with cell-free JEV and intercellular interactions with JEV-associated microglia were applied on microvascular human endothelial cells mimicking receptor-dependent and receptor-independent infection, respectively. Our results demonstrate that human endothelial cells are susceptible to both infection approaches, allowing further productive propagation of JEV including rescue of JEV from microglia. Solely, cytotoxicity towards endothelial cells was exclusively enhanced upon receptor-independent infection. Among candidates responsible for induced-cell death, we identified Tumor Necrosis Factor (TNF) superfamily member 10, TNF-related apoptosis inducing ligand (TRAIL) and TRAIL receptors (TRAILR), as a potential key player in induced cell death. Indeed, TRAIL gene was strongly upregulated in JEV-infected microglia and some genes of TRAILR were greatly expressed in endothelial cells. To summarize, this data suggests that receptor-independent infection of endothelial cells by JEV-associated microglia may contribute to the disruption of the BBB in a TNF-dependent pathway.

## Materials and methods

### Antibodies

For flow cytometry, cells were characterized using fluorescently labelled anti-human CD11b (mouse IgG1κ, clone ICRF44, FITC, BD Biosciences, Franklin Lakes, NJ), anti-human CX_3_CR1 (rat IgG2b, clone 2A9-1, PE, Miltenyi Biotec GmbH, Bergisch Gladbach, Germany) and anti-human CD31 (mouse IgG1κ, clone WM59, AF647, BD Biosciences) antibodies as well as primary anti-human CX_3_CL1 antibody (rabbit polyclonal IgG, clone PA5-23062, Thermofischer Scientific, Waltham, MA) followed by secondary fluorescent-labelled anti-rabbit IgG antibody (donkey, AF647 Abcam, Cambridge, UK).

For nanozoomer, primary anti-human antibodies against CX_3_CL1 and secondary horse anti-mouse IgG peroxidase (MP-7402, ImmPRESS, Vector Laboratories Inc., Newark, CA) were used on human brain sections.

For titration measurements, viral particles were detected using the pan-immune anti-flavivirus antibody (mouse IgG1/IgG2a, clone ATCC-HB-112 D1-4G2-4-15 hybridoma, ATCC, Wesel, Germany).

All antibodies concentrations were optimized in our laboratory according to the methods.

### Preparation of human brain samples and generation of NanoZoomer images

Human brain sections were treated with Bloxall blocking solution (Vector Laboratories, Newark, CA), followed by horse serum. CX_3_CL1 expression was revealed by incubating samples with anti-CX_3_CL1 antibody and subsequent peroxidase enzymatic reaction. Brain sections were then treated with ImmPACT vector Red (Vector Laboratories) before coloration with hematocxylin Mayer (Vector Laboratories). After staining and coloration, slides were then mounted using Vectamount (Vector Laboratories) and left for solidification. Control samples were treated similarly except incubation with primary antibody. Brain sections were analysed using a NanoZoomer 2.0 HT (Hamamatsu Photonics, Hamamatsu, Japan). The image acquisitions were performed with a 20x objective.

### Cell lines culture

Baby Hamster Kidney-21 cells (BHK-21 cells, fibroblasts) ([C-13], ATCC) were cultured in Glasgow’s Minimum Essential Medium (GMEM) (Thermofischer Scientific) supplemented with 10% v/v Fetal Bovine Serum (FBS) (Biowest, Nuaillé, France) and Tryptose Phosphate Broth solution (Sigma-Aldrich, Saint Louis, MO) at 37°C and 5% CO_2_.

Human Dermal Microvascular Endothelial cells (CADMEC/HMVEC cells) (Cell Application Inc., San Diego, CA) were cultured in Endothelial Cell Growth Medium MV supplemented with SupplementMix following manufacturer recommendations (PromoCell GmbH, Heidelberg, Germany) at 37°C and 5% CO_2_.

### Virus preparation and end-point titration

JEV Nakayama isolate (National collection of pathogenic viruses, NCPV, Salibury, UK) was propagated in BHK-21 cells as previously described (Lannes et al., 2017b). Briefly, BHK-21 monolayer cell culture at 80% confluency was infected with JEV suspended in RPMI-1640 GlutaMAX™-I medium (Thermofischer Scientific) supplemented with 2% FBS and cultured until cytopathogenic effects (approx. 36-48 hours). Virus stock suspension was obtained after disruption of remaining cells by freezing and thawing followed by centrifugation at 3000g at 4°C for 30 minutes to eliminate cell debris. Mock antigen was prepared accordingly from uninfected BHK-21 cells and was used as controls in experiments.

Virus titres of virus stocks and experimental supernatants were determined by end-point titration. Briefly, 10-fold serial dilutions of suspension in GMEM supplemented with 10% FBS and Tryptose Phosphate Broth solution were applied on BHK-21 cells at 37°C, 5% CO2. At 36-48 hours post-infection (hpi), intracellular viral particles were detected with the pan-immune anti-flavivirus antibody and subsequent peroxidase enzymatic reaction.

### Treatment of human microvascular endothelial cells with cell-free JEV

In receptor-dependent infection experiments, 5x10^4^ human endothelial cells per well were plated in 24 well plates during 2 hours for adherence. Cells were then treated with Mock or JEV at various multiplicity of infection (MOI) in median tissue culture infectious dose per cell (TCID_50_/cell) in 0.5mL Endothelial Cell Growth Medium MV supplemented with SupplementMix at 37°C and 5% CO_2_. In kinetics experiments, virus adherence was allowed for 2 hours, cells were then intensively washed with warm PBS and cultured in fresh Endothelial Cell Growth Medium MV supplemented with SupplementMix at 37°C and 5% CO_2_ for various time-periods before collection of supernatants for further analysis. In other experiments, inoculum was left on, and cells were cultured for 2 days before collection of cells and supernatants for further analysis.

In supernatants toxicity assessment experiments, 5x10^4^ human endothelial cells were exposed to supernatants of mock and JEV-treated microglia at 6 days post infection (dpi) at a volume of 1:10, meaning 50μL of supernatant plus 450μL of Endothelial Cell Growth Medium MV supplemented with SupplementMix, at 37°C in 5% CO_2_ for 2 days before collection of cells for further analysis.

### Generation of human microglial cells

Human blood monocyte-derived microglia were generated from buffy coats of anonymous healthy donors obtained from the Swiss Red Cross Blood Bank, (Interregionale Blutspende, Bern, Switzerland) using a protocol adapted from (Etemad et al., 2012). Human peripheral blood mononuclear cells (PBMC) were isolated from buffy coat after Ficoll-Paque density gradient centrifugation (1.077 g/L, Amersham Pharmacia Biotech AG, Dubendorf, Switzerland). Monocytes were enriched based on CD14^+^ positive selection using selection columns and magnetic sorting system (Miltenyi Biotech GmbH). CD14^+^ cells per well were cultured at a concentration of 0.5x10^6^ cells/mL in RPMI-1640 GlutaMAX™-I medium supplemented with antibiotic/antimycotic and bioactive human recombinant granulocyte macrophage colony-stimulating factor (GM-CSF) (10ng/mL), macrophage colony-stimulating factor (M-CSF) (10ng/mL), nerve growth factor (NGF)-β (10ng/mL) and CC chemokine ligand 2 (CCL2) (50ng/mL) (all purchased from Miltenyi Biotech GmbH), at 37°C and 5% CO_2_ for 7 days. Half of the medium, containing the concentration for cytokines/chemokines at final volume, was renewed at days 3 and 6 of culture.

In transcriptomics studies, 2x10^6^ CD14^+^ cells were plated in 6 well plates. In co-culture studies, 2.5x10^5^ CD14^+^ cells were plated in 24 well plates.

### Treatment of human microglia cells with JEV

In transcriptomic studies, differentiated human microglia cells were treated with Mock or JEV (at a MOI of 1 TCID_50_/cell) in 4mL RPMI-1640 GlutaMAX™-I medium (Thermofischer Scientific) at 37°C and 5% CO_2_ for 24 hours. Then, cells were used in further analysis.

In co-culture studies, differentiated human microglia cells were treated with Mock or JEV (at a MOI of 10 TCID_50_/cell) in 0.5mL RPMI-1640 GlutaMAX™-I medium at 37°C and 5% CO_2_ for 6 days. This led to the generation JEV-associated microglia. In parallel, supernatants were collected for further analysis.

### Preparation of RNA and generation transcriptome library

Approximately 2x10^6^ microglia per condition and 3x10^5^ microvascular endothelial cells were used for total RNA preparation. RNA from cells were extracted using TriZOL reagent (Life Technologies, Zug, Switzerland) and Tri reagents (Molecular Research Center, Inc., Cincinnati, OH), both supplemented with glycogen (Life Technologies), for human microglia cells and microvascular endothelial cells, respectively. Then, further steps of RNA extraction were performed. RNA was dissolved in RNase-free water and tested for quality and quantity with the 2200 TapeStation system (Agilent technologies, Santa Clara, CA). After validation of RNA samples quality, transcriptome libraries were generated by Novogene Co Ltd (Beijing, China) using the following parameters: Human mRNA sequencing (WBI-Quantification), Illumina HiSeqPE150 sequencing platform (PE150, Q30>85%), mRNA library preparation (polyA enrichment). The Reference Genome and Version was GRCh38.

### Co-culture of JEV-associated microglia and human microvascular endothelial cells

After collection of supernatants, JEV-associated microglia were washed 5 times with cold PBS. Both supernatant and last washing were confirmed negative for infectious JEV by end-point titration. Subsequently, 5x10^4^ human endothelial cells in 0.5mL Endothelial Cell Growth Medium MV supplemented with SupplementMix were added to the latter JEV-associated microglia and co-culture was allowed for 2 days at 37°C in 5% CO_2_. Supernatants and cells were collected for further analysis.

In targeted co-cultures studies, CX_3_CR1 antagonist could be added at various concentrations (Axon Biochemicals, Groningen, The Netherlands) suspended in DMSO (Thermofischer Scientific) and DMSO was used as control.

### Cytotoxicity assay

Cytotoxicity was evaluated by measuring the apoptotic cell content using Annexin-V-FITC kit following manufacturer recommendation (Miltenyi Biotech GmbH). Briefly, cells were gently detached using EDTA (10mM) in PBS buffer on ice and processed using manufacturer Annexin-V buffer for Annexin-V reaction and washings. In addition, cells were fixed with PFA at 4% prepared in the manufacturer Annexin-V buffer to ensure maintained binding of Annexin-V. Finally, Annexin-V^+^ cells were analysed by flow cytometry.

### Flow cytometry

Cells were analysed using alternatively the following multi-colour flow cytometry instruments: FACSCanto II (BD Biosciences), BD Accuri C6 Plus (BD Biosciences) and Aurora (Cytek Biosciences, Fremont, CA). Data were analysed using FlowJo Software (Data analysis Software, Ashland, OR).

### Statistical analysis

Significant differences were determined with GraphPad Prism 6 software (GraphPad software Inc., La Jolla, CA) using the unpaired Mann-Whitney test (p<0.05), the unpaired t-test (p<0.05) or the 2-way ANOVA test (p<0.05).

## Results

### Human microvascular endothelial cells are susceptible to both receptor-dependent and receptor-independent infections by JEV

**Figure 1:**
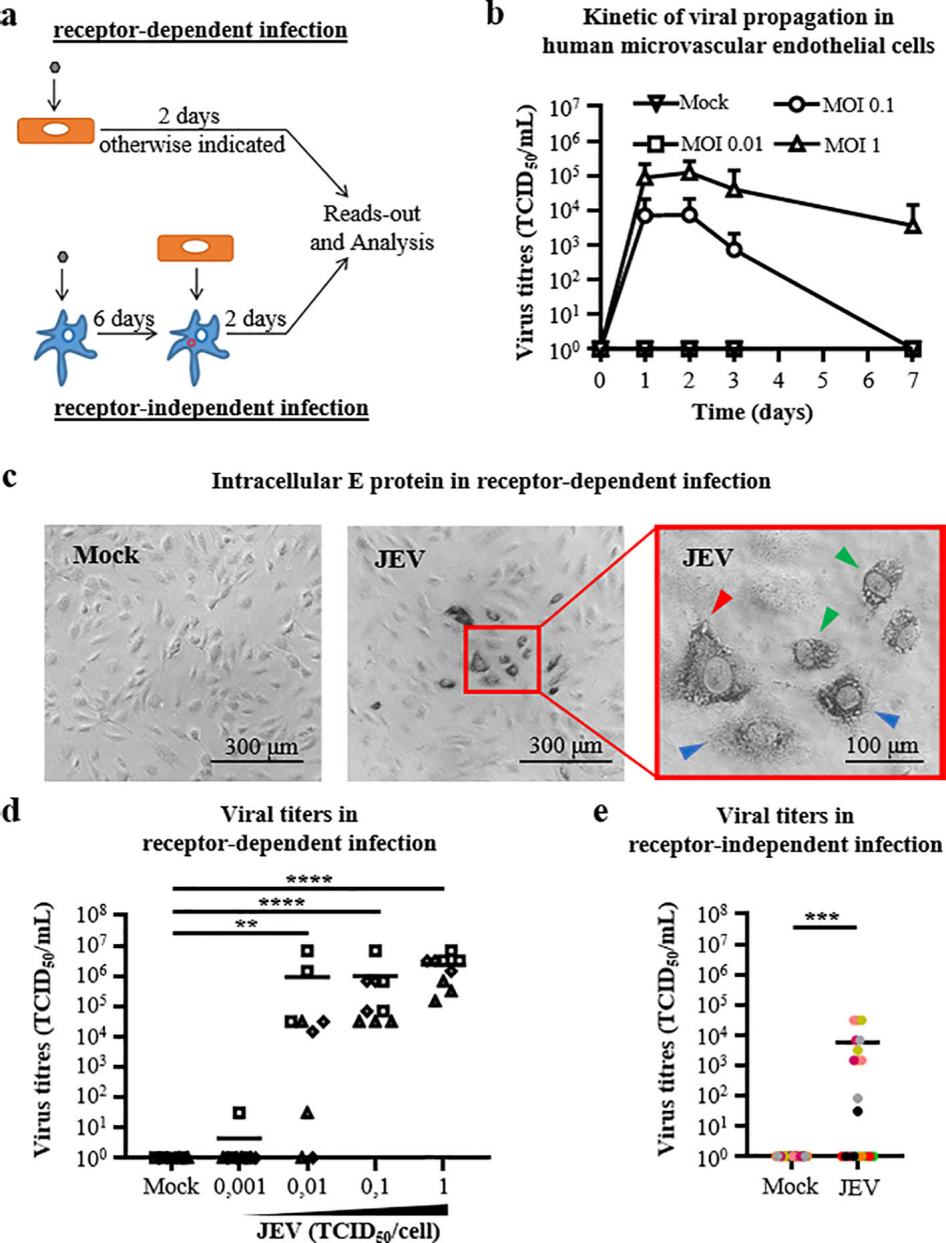
Virus propagation in receptor-dependent and receptor-independent infection of human microvascular endothelial cells. **(a)** Schematic representation of infection of microvascular endothelial cells (upper panel) in a receptor-dependent manner of using cell-free JEV and (lower panel) in a receptor-independent manner using JEV-associated microglia. Microvascular endothelial cells are in orange, microglia in blue, cell-free JEV in grey and JEV-associated microglia in red. **(b)** Curve lines representing kinetic studies of virus titres in supernatants of receptor-dependent infection of microvascular endothelial cells with mock and cell-free JEV at indicated multiplicity of infection (MOI) in TCID_50_/cell. Data are of 3 independent experiments with each condition performed in triplicate. The symbol represents the mean value; the error bars the standard deviation. **(c)** Representative light microscopy images of intracellular E protein detection (in dark) of mock and cell-free JEV-treated microvascular endothelial cells with a MOI of 1 TCID_50_/cell at 2 dpi. A higher magnification is shown in the red frame. Green arrows show a wide distribution in the cytoplasm, blue arrow a perinuclear location and red arrow an inner cytoplasmic membrane wall location of E proteins. Scale bar is indicated. **(d, e)** Scatter dot plot representing virus titres in supernatants of **(d)** receptor-dependent infection with mock and cell-free JEV at indicated MOI and in **(e)** receptor-independent infection with mock and JEV-associated microglia, with a MOI of 10 TCID_50_/cell for 6 days. Data are of independent experiments with each condition performed in triplicate, the solid line is the mean value. **(d)** Each symbol represents an experiment (#3) and **(e)** each colour represents a blood donor (#7). Asterisks show significant differences using the unpaired Mann-Whitney test (*p<0.05; **p<0.01; ***p<0.001; ****p<0.0001).

Microvascular endothelial cells are known to be productive JEV propagators (Shwetank et al., 2013). Therefore, we first assessed the susceptibility of the human microvascular endothelial cell line to JEV in a multiple dose and kinetic study in a receptor-dependent infection approach (**Figure 1a**, upper panel). Here, MOIs of 0.01, 0.1 and 1 TCID_50_/cell were used with cell-free JEV and virus binding was allowed for 2 hours before the inoculum was intensively washed off and fresh medium was added for various incubation periods. Supernatants were collected at 0-, 1-, 2, 3- and 7-dpi. Mock controls underwent similar process. Virus titres were measured in supernatants and peaked at 2 dpi with virus titres of 7.4x10^3^ (± 1.4x10^4^) TCID_50_/ml and 1.2x10^5^ (± 1.5x10^5^) TCID_50_/ml for MOI of 0.1 and 1 TCID_50_/cell, respectively. Virus titres of 3.7x10^3^ (± 1x10^4^) TCID_50_/ml were detected at 7 dpi with a MOI of 1 TCID_50_/cell only. However, using a MOI of 0.01 TCID_50_/cell condition failed to produce infectious JEV (**Figure 1b**). In conclusion, human microvascular endothelial cells are productively infected by JEV with an optimum incubation time of 2 days. Therefore, an incubation time of 2 days was defined for JEV interactions with human microvascular endothelial cells in further experiments. Thence, intracellular E protein detection was further analysed with the microscope in receptor-dependent infection of human microvascular endothelial cells. Here, cells were infected with cell-free JEV at a MOI of 1 TCID_50_/cell for 2 days, without washing off the inoculum. Intracellular E protein was confirmed in a cell cluster fashion. In details, E protein was found widely distributed over in the cytoplasm, concentrated at the perinuclear location and/or concentrated at the inner wall of the cytoplasmic membrane (**Figure 1c**). Then, efficacy of receptor-dependent infection of human microvascular endothelial cells by JEV without removal of the inoculum was tested in a multiple dose study. In this approach, mock and cell-free JEV were applied on human endothelial cells for 2 days using MOIs from 0.001 to 1 TCID_50_/cell. As a result, significant virus titres in supernatants were observed at MOIs of 0.01 TCID_50_/cell and higher. Importantly, MOIs of 0.1 and 1 TCID_50_/cell showed accurate viral production in all replicates, with virus titres of 1x10^6^ (± 2.2x10^6^) TCID_50_/ml and 2.1x10^6^ (± 1.5x10^6^) TCID_50_/ml, respectively (**Figure 1d**). To conclude, human microvascular endothelial cells are susceptible to JEV with potent viral propagation abilities with various sensitivities in various receptor-dependent infection approaches.

In parallel, the susceptibility of human microvascular endothelial cells in receptor-independent infection was assessed. To this end, human monocyte-derived microglia were treated with mock and JEV at a MOI of 10 TCID_50_/cell for 6 days leading to the generation of JEV-associated microglia. In addition, no infectious JEV was detected in supernatants of such culture conditions (data not shown). At 6dpi, mock- and JEV-associated microglia were co-cultured with human microvascular endothelial cells for 2 days (**Figure 1b**, lower panel). After 2 days of co-culture (dcc), virus titres in supernatant were of 5.8x10^3^ (± 1.1x10^4^) TCID_50_/ml. However, discrepancies between donors were notable in the efficacy in virus rescue upon intercellular virus transmission from JEV-treated microglia to endothelial cells (**Figure 1e**). Overall, human microvascular endothelial cells are susceptible to JEV with limited viral propagation abilities in a receptor-independent infection approach.

### Receptor-independent infection of human microvascular endothelial by JEV is cytotoxic

Although the mechanism behind neuroinvasion remains to be fully elucidated, JEV invades the human CNS by crossing the blood-brain barrier (BBB) (Filgueira and Lannes, 2019). JEV transmigrate across human microvascular endothelial cells inducing the expression of pro-apoptotic proteins (Al-Obaidi et al., 2017). Thus, we assessed the cytotoxicity of receptor-dependent and receptor-independent infection approaches by analysing the exposure of phosphatidylserine on the cell surface using flow cytometry analysis of Annexin-V staining.

**Figure 2:**
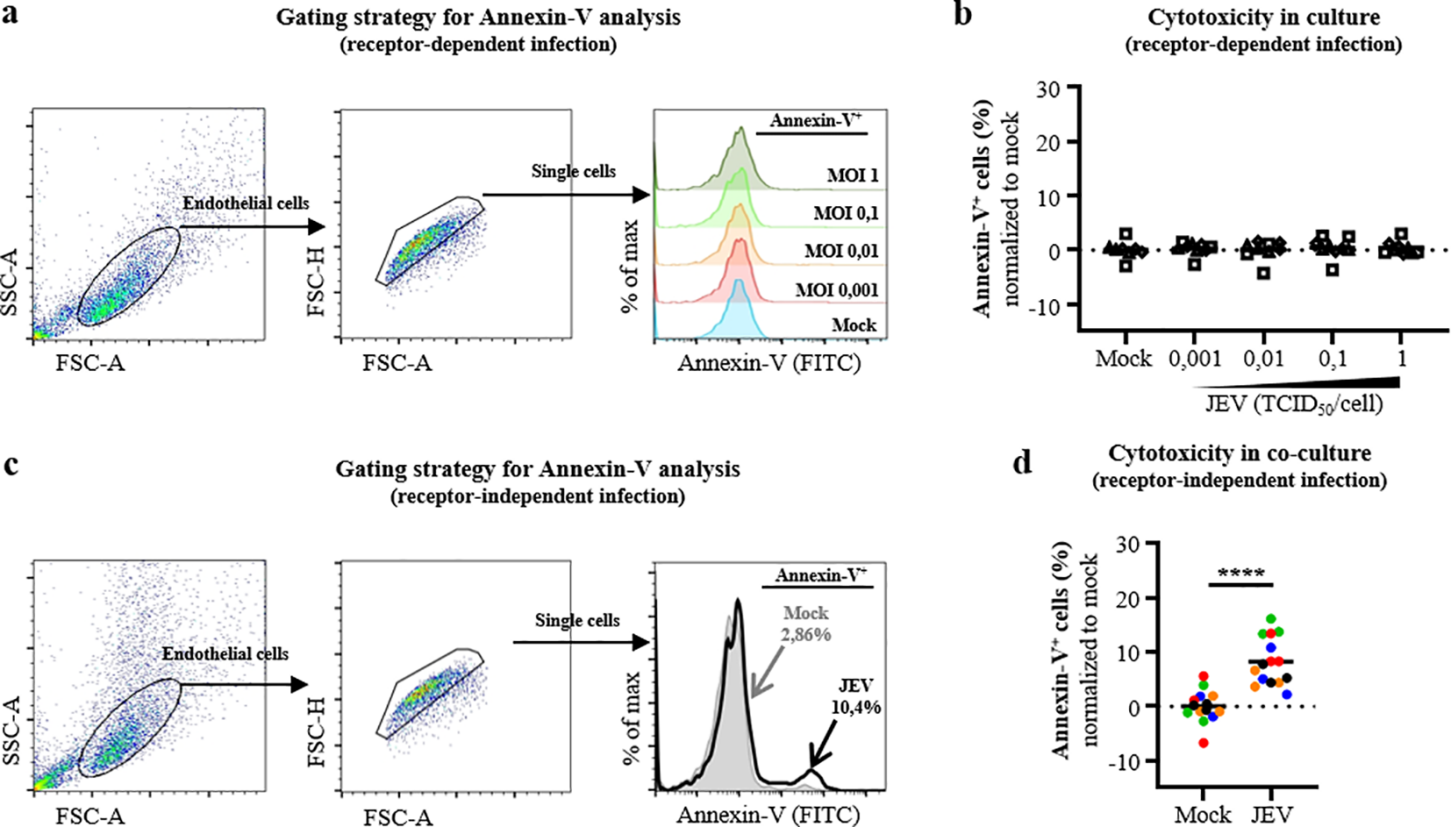
Cell death of human microvascular endothelial cells in receptor-dependent and receptor-independent infection. **(a)** Representative gating strategy for flow cytometry analysis of Annexin-V staining in receptor-dependent infection of microvascular endothelial cells with mock and cell-free JEV at indicated MOI infection. Selected endothelial cells based on FSC/SSC profile after debris exclusion (left panel) and single cells selection (middle panel) are shown in pseudo-plot representation. Subsequent histogram plot shows Annexin-V^+^ microvascular endothelial cells (right panel). **(b)** Scatter dot plot representing the frequencies of Annexin-V^+^ single microvascular endothelial cells in a receptor-dependent infection, as described in **(a)**. **(c)** Representative gating strategy for flow cytometry analysis of Annexin-V staining in receptor-independent infection of microvascular endothelial cells with mock- and JEV-associated microglia, with a MOI of 10 TCID_50_/cell for 6 days. Selected endothelial cells based on FSC/SSC profile after debris exclusion (left panel) and single cells selection (middle panel) are shown in pseudo-plot representation. Subsequent histogram plot shows frequencies of Annexin-V^+^ microvascular endothelial cells (right panel). **(d)** Scatter dot plot representing the frequencies of Annexin-V^+^ single microvascular endothelial cells in a receptor-independent infection, as described in **(c)**. Data are of independent experiments with each condition performed in triplicate, the solid line is the mean value and the dashed line is the baseline (y=0%). **(b)** Each symbol represents an experiment (#3) and **(d)** each colour represents a blood donor (#5). Asterisks show significant differences using the unpaired t-test (*p<0.05; **p<0.01; ***p<0.001; ****p<0.0001).

In order to evaluate the cytotoxicity in receptor-dependent infection, human microvascular endothelial cells were treated in dose study using MOIs from 0.001 to 1 TCID_50_/cell, as previously described in conditions of not washing out the inoculum. At 2dpi, cells were assessed for early apoptosis. Endothelial cells were identified based on their higher Forward scatter/Side scatter (FSC/SSC) profile and subsequent analysis of Annexin-V was performed on single cells (**Figure 2a**). In these conditions, JEV-infected endothelial cells did not show any differences in Annexin-V levels compared to mock control, independently to the MOI applied (**Figure 2b**).

Likewise, the cytotoxicity in a receptor-independent infection was measured in as previously described co-cultures. Mock and JEV-associated microglia, for 6 days with a MOI of 10 TCID_50_/cell, were co-cultured with human microvascular endothelial cells for 2 days. First, supernatants of JEV-treated microglia at 6dpi did not induce any early apoptosis on human endothelial cells, minimizing cytotoxic effects from possible inflammatory soluble factors secreted by JEV-pulsed microglia (**Supplementary Figure 1**). Then, cells at 2dcc were assessed for early apoptosis. Due to overlapping in CD11b, CX_3_CR1 and CD31 expressions between human microglia and human endothelial cells, these markers were insufficient to discriminate the two cell types. However, endothelial cells displayed a higher FSC/SSC profile than human microglia (**Supplementary Figure 2a**). Therefore, FSC/SSC profile was used as a discriminating parameter in further analysis and subsequent analysis of Annexin-V was performed on single microvascular endothelial cells (**Figure 2c**). Here, JEV-associated microglia induced significant increase of Annexin-V staining of 8.2 (± 4.3) % of endothelial cells compared to mock control (**Figure 2d**). In parallel, a significant increase of Annexin-V staining of 6.7 (± 6.3) % was observed in human microglial cells at 2dcc (**Supplementary Figure 2c**). Nevertheless, the FSC/SSC profile of the microglia population could overlap with the one of debris from endothelial cells (**Figure 2a**, **Supplementary Figure 2a**, left panels) and the majority of events stained for Annexin-V in mock controls in the microglial analysis (<70%, **Supplementary Figure 2b**). To conclude, JEV induces cytotoxicity to human microvascular endothelial cells in receptor-independent infection but not in receptor-dependent infection.

### Cytotoxic effects by JEV-associated microglia in receptor-independent infection is independent to CX_3_CR1-CX_3_CL1 interactions

Microglia and endothelial cells largely interact in various channels (Sheikh et al., 2019). Membrane-bound CX_3_CL1, expressed by endothelial cells, may serve as adhesion molecule promoting intercellular interaction and endothelial cell injury in diseases including in viral infection (Zhang et al., 2024). Since the CX_3_CR1-CX_3_CL1 axis may be critical in viral transmission between JEV-infected microglia to a target cell (Lannes et al., 2019), we analysed virus rescue and cytotoxicity towards cells in receptor-independent infection after the blockade of CX_3_CR1-CX_3_CL1 interactions.

**Figure 3:**
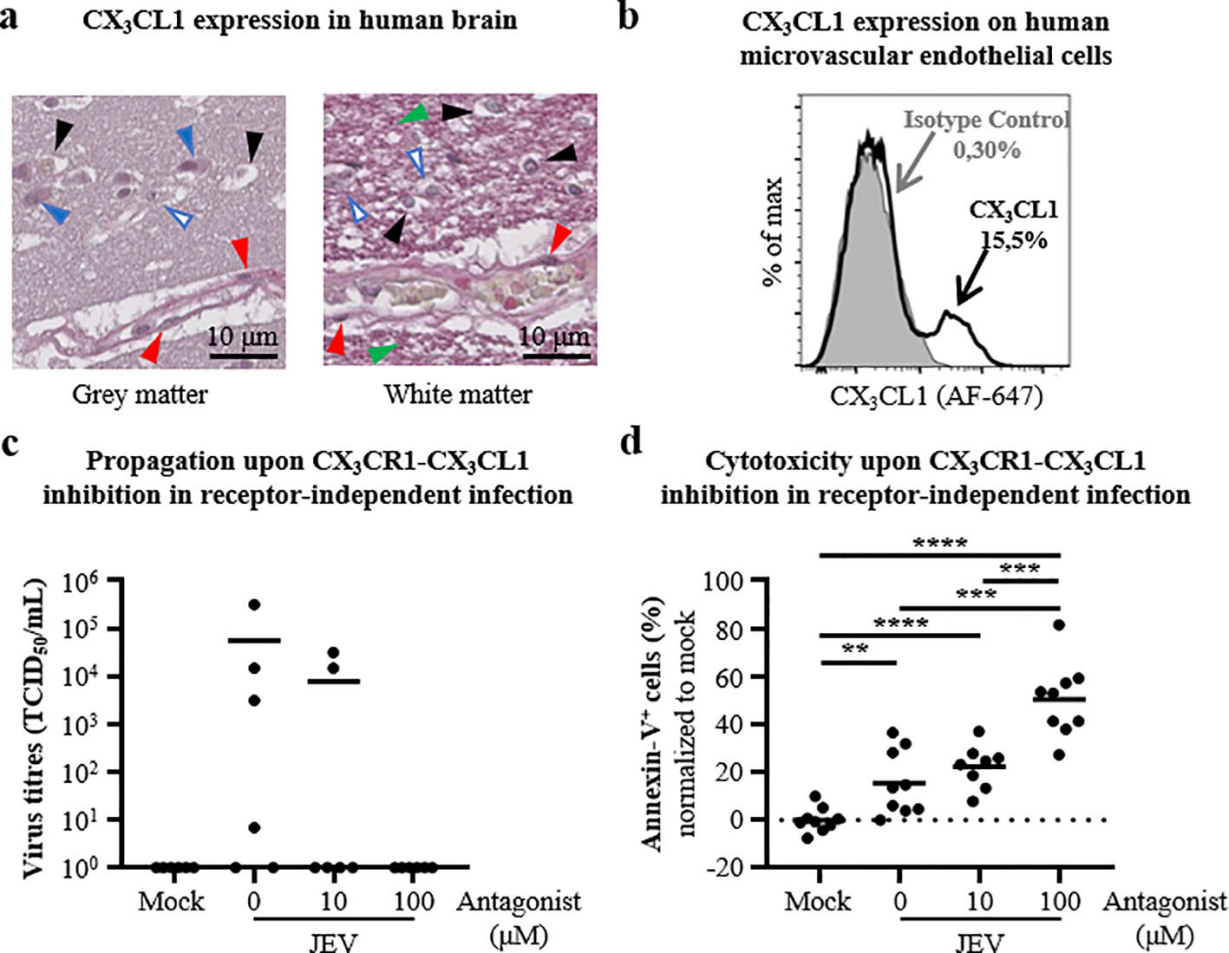
Impact of CX_3_CR1-CX_3_CL1 inhibition in receptor-independent infection. **(a)** Representative micrographs of CX_3_CL1 expression in the grey matter (left panel) and the white matter (right panel) in the frontal lobe of the human brain. In vascular areas, red arrows highlight CX_3_CL1 expression in endothelial cells of blood vessels. In cerebral matter, green arrows CX_3_CL1 expression out of cellular bodies, black arrows CX_3_CL1 non-expressing cells, and solid and open blue arrows show CX_3_CL1 expressing cells with high and low intensity, respectively. Scale is indicated. **(b)** Representative histogram plot of flow cytometry analysis showing frequencies of CX_3_CL1^+^ microvascular endothelial cells at steady state including the isotype control (filled grey), after single cell selection. **(c)** Scatter dot plot representing virus titres in supernatants of receptor-independent infection of microvascular endothelial cells with mock and JEV-associated microglia, with a MOI of 10 TCID_50_/cell for 6 days, in presence of DMSO and indicated concentration of CX_3_CR1 antagonist. **(d)** Scatter dot plot representing the frequencies of Annexin-V^+^ single microvascular endothelial cells in a receptor-independent infection of microvascular endothelial cells mock and JEV-associated microglia, with a MOI of 10 TCID_50_/cell for 6 days, in presence of DMSO and indicated concentration of CX_3_CR1 antagonist, as gated in **Figure 2c**. **(c, d)** Data are of **(c)** 2 and **(d)** 3 independent experiments (# of blood donors) with each condition performed in triplicate, the solid line is the mean value and **(d)** the dashed line is the baseline. Statistics are calculated with **(d)** the unpaired t-test (*p<0.05; **p<0.01; ***p<0.001; ****p<0.0001).

In order to verify whether the CX_3_CR1-CX_3_CL1 axis may apply, we first assessed the expression of CX_3_CL1 by endothelial cells in the human brain and in cell culture. As expected, CX_3_CL1 was expressed by endothelial cells of blood vessel walls in both the grey and white matter of human brain sections of the frontal lobe. In the grey matter, CX_3_CL1 was found in cells at high and low intensities, resembling neuronal cell bodies and astrocytes respectively (Hulshof et al., 2003). In parallel, other cells, probably microglia (Hulshof et al., 2003), and pericellular areas were free from CX_3_CL1 expression. In the white matter, some cells were CX_3_CL1^+^ whereas others were CX_3_CL1^-^, probably astrocytes and microglia respectively (Hulshof et al., 2003). In addition, pericellular areas demonstrated high intensity of CX_3_CL1 (**Figure 3a**). In cell culture, around 15.5% of human microvascular endothelial cells exhibited CX_3_CL1 expression at steady state (**Figure 3b**). In further receptor independent infection experiments using 10 and 100 μM of antagonist for CX_3_CR1, human microglial cells at 2dcc displayed reduced cell death levels in presence of antagonist in a dose dependent fashion (**Supplementary Figure 2d**). Nevertheless, most important is that virus rescue was not limited by the use of an antagonist for CX_3_CR1 at 10μM and cytotoxicity towards endothelial cells was unaffected. Also, despite the fact that virus rescue was completely abrogated by using 100 μM of the antagonist for CX_3_CR1, the cytotoxicity towards endothelial cells was dramatically enhanced at values of 50.2 (± 15.7) % (**Figures 3c, d**). Therefore, the CX_3_CR1-CX_3_CL1 axis is not a relevant therapeutic target in limiting virus rescue and cell death during intercellular communication between microglial and endothelial cells.

### Identification of candidates for cell death towards JEV-treated microglia and human microvascular endothelial cells

In order to identify possible molecular mediators of cell death in receptor-independent infection, we performed transcriptomics analysis of cell death associated genes such as caspase genes, programmed cell death (PD) genes and TNF genes (**Table 1**) in JEV-treated microglia and steady state endothelial cells in culture.

**Table 1:** List of cell death associated genes including programmed cell death (PD), caspase and Tumor necrosis factor members.

Family name	Gene name	Protein Name	Ensembl gene ID
Programmed	*PDCD1*	Programmed cell death protein 1 (PD-1)	ENSG00000188389
Cell Death	*CD274*	Programmed death ligand 1 (PD-L1)	ENSG00000120217
	*PDCD1LG2*	Programmed cell death 1 ligand 2 (PD-L2)	ENSG00000197646
Caspases	*CASP2*	Caspase 2	ENSG00000106144
	*CASP8*	Caspase 8	ENSG00000064012
	*CASP9*	Caspase 9	ENSG00000132906
	*CASP10*	Caspase 10	ENSG00000003400
	*CASP3*	Caspase 3	ENSG00000164305
	*CASP6*	Caspase 6	ENSG00000138794
	*CASP7*	Caspase 7	ENSG00000165806
	*CASP1*	Caspase 1	ENSG00000137752
	*CASP4*	Caspase 4	ENSG00000196954
	*CASP5*	Caspase 5	ENSG00000137757
	*CASP12*	Caspase 12	ENSG00000204403
	*CASP14*	Caspase 14	ENSG00000105141
TNF receptors	*TNFRSF1A*	TNF receptor (TNFR) 1	ENSG00000067182
	*TNFRSF1B*	TNFR2	ENSG00000028137
	*LTBR*	Lymphotoxin β receptor (LTBR)	ENSG00000111321
	*TNFRSF4*	AX40	ENSG00000186827
	*CD40*	CD40	ENSG00000101017
	*FAS*	Fas receptor (FasR)	ENSG00000026103
	*TNFRSF6B*	Decoy receptor 3	ENSG00000243509
	*CD27*	CD27	ENSG00000139193
	*TNFRSF8*	CD30	ENSG00000120949
	*TNFRSF9*	4-1BB	ENSG00000049249
	*TNFRSF10A*	TNF-related apoptosis-inducing ligand receptor 1 (TRAILR1)	ENSG00000104689
	*TNFRSF10B*	TRAILR2	ENSG00000120889
	*TNFRSF10C*	TRAILR3	ENSG00000173535
	*TNFRSF10D*	TRAILR4	ENSG00000173530
	*TNFRSF11A*	Receptor activator of nuclear factor κ-B (RANK)	ENSG00000141655
	*TNFRSF11B*	Osteoprotegerin	ENSG00000164761
	*TNFRSF12A*	TNF-related weak inducer of apoptosis receptor (TWEAKR)	ENSG00000006327
	*TNFRSF13B*	Transmembrane activator and CAML interactor (TACI)	ENSG00000240505
	*TNFRSF13C*	B-cell activating factor receptor (BAFF-R)	ENSG00000159958
	*TNFRSF14*	TNFRSF14	ENSG00000157873
	*NGFR*	TNFRSF16	ENSG00000064300
	*TNFRSF17*	TNFRSF17	ENSG00000048462
	*TNFRSF18*	TNFRSF18	ENSG00000186891
	*TNFRSF19*	TNFRSF19	ENSG00000127863
	*RELT*	TNFRSF19L	ENSG00000054967
	*TNFRSF21*	TNFRSF21	ENSG00000146072
	*TNFRSF25*	TNFRSF25	ENSG00000215788
	*EDA2R*	TNFRSF27	ENSG00000131080
	*EDAR*	EDAR	ENSG00000135960
TNF ligands	*LTA*	Lymphotoxin α	ENSG00000226979
	*TNF*	TNF-α	ENSG00000232810
	*LTB*	Lymphotoxin β	ENSG00000227507
	*TNFSF4*	OX40 ligand	ENSG00000117586
	*CD40LG*	CD40 ligand	ENSG00000102245
	*FASLG*	Fas ligand	ENSG00000117560
	*CD70*	CD27 ligand	ENSG00000125726
	*TNFSF8*	CD30 ligand	ENSG00000106952
	*TNFSF9*	CD137 ligand	ENSG00000125657
	*TNFSF10*	TRAIL	ENSG00000121858
	*TNFSF11*	RANK ligand	ENSG00000120659
	*TNFSF12*	TWEAK	ENSG00000239697
	*TNFSF13*	A proliferation inducing ligand (APRIL)	ENSG00000161955
	*TNFSF13B*	BAFF	ENSG00000102524
	*TNFSF14*	LIGHT	ENSG00000125735
	*TNFSF15*	Vascular endothelial growth inhibitor (VEGI)	ENSG00000181634
	*TNFSF18*	TNF sperfamily member 18	ENSG00000120337
	*EDA*	Ectodyplasin A	ENSG00000158813

The list details family name, gene name with corresponding protein name and linked human Ensembl gene identification (ENSG) in GRCh38 reference bank.

**Figure 4:**
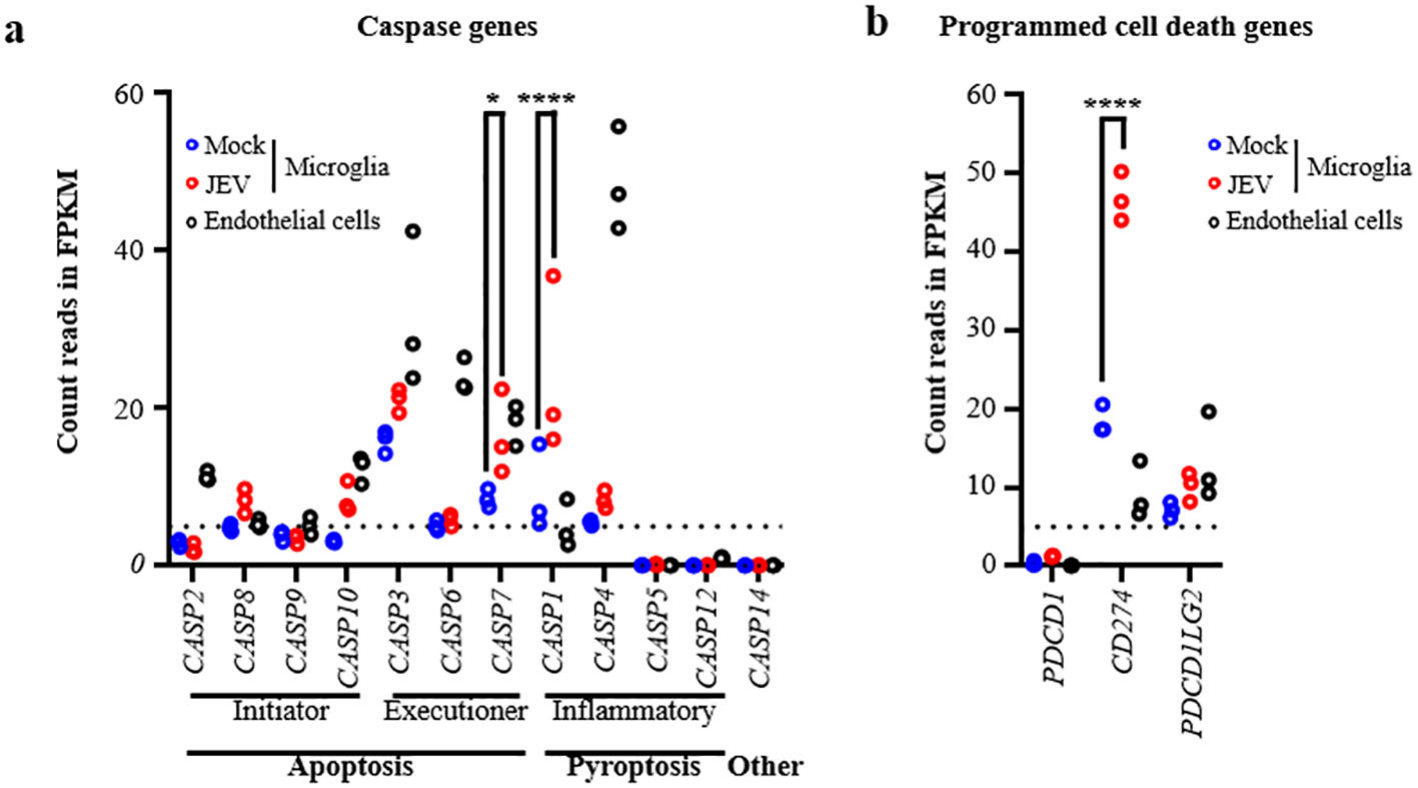
Transcriptomic analysis of caspase and programmed cell death genes in human microglia and human microvascular endothelial cells. **(a,b)** Scatter dot plot representing count reads in Fragments Per Kilobase of transcript per Million mapped reads (FPKM) of human **(a)** caspase genes subclassified into types and involved processes and **(b)** programmed cell death ligands and receptors genes, after transcriptomic analysis in mock- and JEV-treated microglia and in human microvascular endothelial cells at steady state. Data are of 3 independent experiments the dashed line is the baseline (= 5 FPKM). For microglia model, each symbol represents a blood donor in which cells have treated with mock or JEV. For human microvascular endothelial cells, each symbol represents a batch of the cell culture. Statistics are calculated with the 2-way ANOVA test (*p<0.05; **p<0.01; ***p<0.001).

Caspases are a family of cysteine protease enzymes whose intracellular activity is involved in programmed cell death with functions in apoptosis. Among caspases candidates, mock-treated human microglia constitutively expressed mRNA for genes *CASP3*, *CASP6*, *CASP7* and *CASP1*. Compared to mock, JEV treatment induced significant up-regulation for *CASP7* and *CASP1* gene expressions in human microglia by 8.0 (± 5.4) Fragments Per Kilobase of transcript per Million mapped reads (FPKM) and 14.8 (± 11.2) FPKM, respectively. Although not significant, treatment with JEV tended to upregulate mRNA expression of *CASP8*, *CASP10*, *CASP3* and *CASP4*. In parallel, endothelial cells constitutively expressed mRNA for genes *CASP2*, *CASP10*, *CASP6*, *CASP7* and *CASP4* (**Figure 4a**). Overall, cell death of JEV-pulsed microglia and microvascular endothelial cells may involve caspases activity such as caspase 7 in apoptosis and caspase 1 via the inflammasome in necroptosis and/or pyroptosis.

Programmed cell death molecules are a family of one exclusive receptor and two ligands from which interactions are involved in apoptosis. First, mRNA for the PD receptor gene, *PDCD1*, remained under baseline level in mock-treated human microglia and undetectable in steady state endothelial cells (**Figure 4b**). Second, Mock-treated human microglia constitutively expressed mRNA for both genes of PD ligands, *CD274* and *PDCD1LG2*. Upon JEV treatment, *CD274* gene expression was significantly increased by 28.5 (± 3.1) FPKM and *PDCD1LG2* mRNA levels were slightly upregulated compared to mock. Interestingly, *CD274* and *PDCD1L2* mRNA were constitutively in expressed steady state endothelial cells (**Figure 4b**). Therefore, cell death of JEV-treated microglia and microvascular endothelial cells may not be dependent to PD activity.

**Figure 5:**
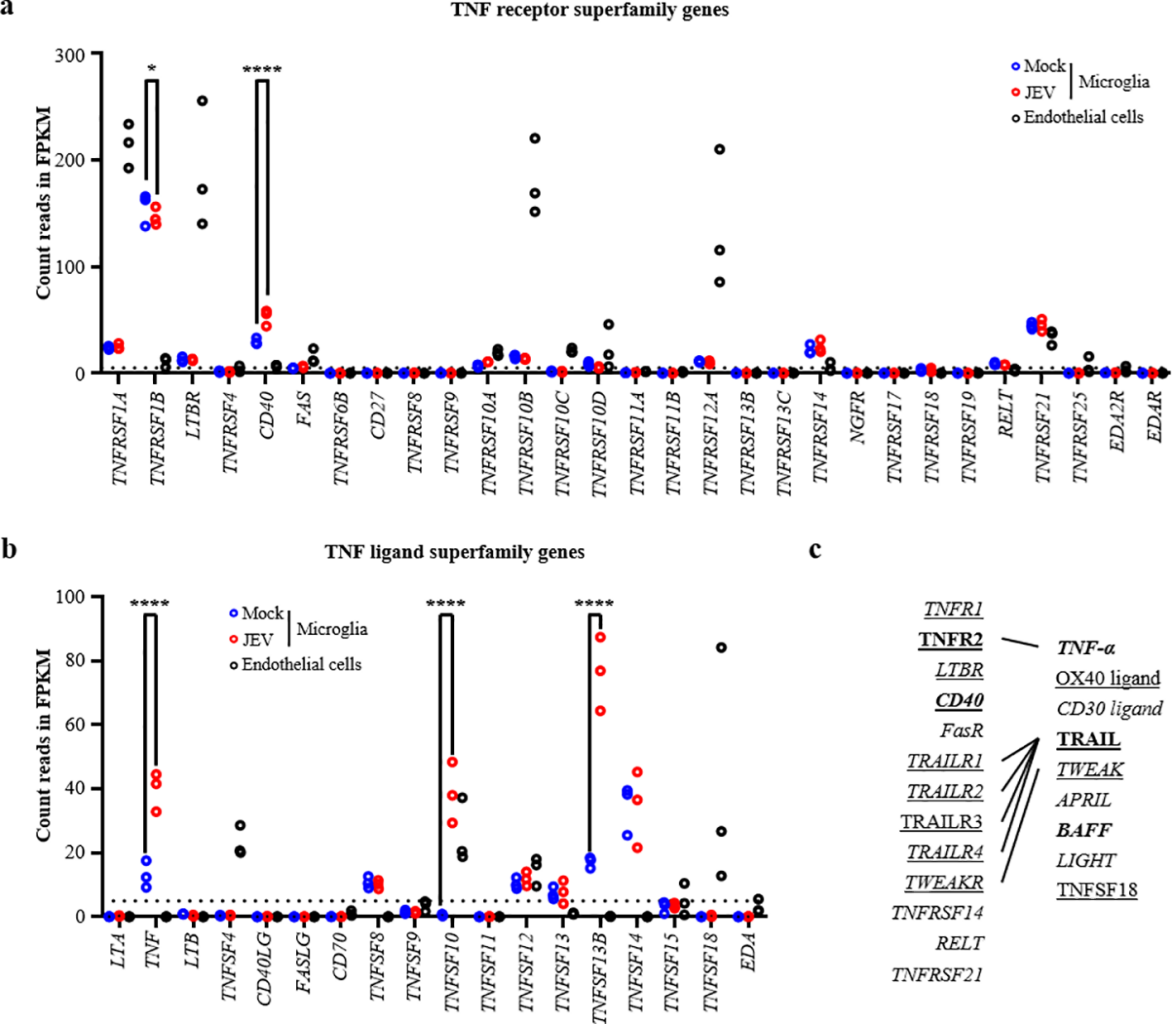
Transcriptomic analysis of TNF receptors and ligands superfamily genes in human microglia and human microvascular endothelial cells. **(a, b)** Scatter dot plot representing gene count in FPKM of human **(a)** TNF receptors and **(b)** ligands superfamily genes after transcriptomic analysis in mock- and JEV-treated microglia and in human microvascular endothelial cells at steady state. Data are of 3 independent experiments the dashed line is the baseline (= 5 FPKM). For microglia model, each symbol represents a blood donor in which cells have treated with mock or JEV. For human microvascular endothelial cells, each symbol represents a batch of the cell culture. Statistics are calculated with the 2-way ANOVA test (*p<0.05; **p<0.01; ***p<0.001). **(c)** Schematic summary of proteins interactions between TNF superfamily ligands with TNF superfamily receptors. Genes of corresponding protein names detailed in **Table 1** and expressed and/or modulated in **(a, b)**, are highlighted: underlined are genes expressed by human microvascular endothelial cell; in italic are genes expressed mock-treated microglia; and in bold are genes regulated by JEV in human microglia.

The TNF super-families are two large families of TNF receptors and ligands involved in cell death processes such as apoptosis if interactions between receptor with its ligand happen. Among TNF receptors, mock-treated human microglia constitutively expressed mRNA for *TNFRSF1A*, *TNRFS1B*, *LTBR*, *CD40*, *TNFRSF10A*, *TNFRSF10B*, *TNFRSF10D*, *TNFRSF12A*, *TNFRSF14, RELT* and *TNFRSF21*. Of relevance, JEV-treated microglia significantly up-regulated the expression of mRNA for *CD40* by 23.0 (± 7.6) FPKM and significantly down-regulated expression of mRNA for *TNRFS1B* by 8.7 (± 8.4) FPKM, compared to mock. In parallel, endothelial cells constitutively expressed mRNA for *TNFRSF1A*, *TNFRSF1B*, *LTBR*, *CD40*, *FAS*, *TNFRSF10A*, *TNFRSF10B*, *TNFRSF10C*, *TNFRSF10D*, *TNFRSF12A* and TNFRSF*21* (**Figure 5a**). Focusing on TNF ligands, mock-treated microglia constitutively expressed mRNA for *TNF*, *TNFSF8*, *TNFSF12*, *TNFSF13*, *TNFSF13B* and *TNFSF14*. Importantly, JEV-infected microglia significantly up-regulated the expression of mRNA for genes *TNF*, *TNFSF10* and *TNFSF13B* with differences of 26.7 (± 6.1) FPKM, 38.0 (± 9.5) FPKM and 59.2 (± 11.6) FPKM, respectively. In parallel, endothelial cells constitutively expressed mRNA for the genes *TNFSF4*, *TNFSF10*, *TNFSF12*, *TNFSF15* and *TNFSF18* (**Figure 5b**).

In order to better clarify the impact of TNF super-families for cell death in co-culture system, **Table 1** relates gene names with corresponding protein names as used in **Figure 5c** and further description. **Figure 5c** recapitulates protein interactions between TNF receptors and respective ligands in correlation with previously described transcriptomics analysis. On one hand, the focus on autocrine effect highlighted two potential TNF candidates potentially affecting microglia but none affecting endothelial cells. First, TNF-α is known to interacts with its receptor TNF receptor 2, both corresponding genes being found in microglia (**Figure 5c**). Despite reduced mRNA expression of TNF receptor 2 upon JEV treatment, endogenous effects may occur in microglia. Second, TRAIL can interact with the TRAILR1, TRAILR2 and TRAILR4, whose corresponding genes are found in JEV-infected microglia. Therefore, endogenous activity of TRAIL via specific TRAILR may induce potential JEV-microglial cell death (**Figure 5c**). In parallel, none of the ligands found in endothelial cells could interact with their corresponding receptors on endothelial cells, excluding endogenous activity of TNF superfamily members (**Figure 5c**). On another hand, the focus on paracrine effect highlighted two potential TNF candidates potentially affecting endothelial cells death induced by JEV-treated microglia. First, TNF-α in JEV-infected microglia may interact with TNF receptor 2 found in endothelial cells leading to endothelial cell death but with limited impact due to low levels of receptor’s mRNA in endothelial cells. Second, TRAIL found in JEV-treated microglia may interact with the TRAIL receptors TRAILR1, TRAILR2, TRAILR3 and TRAILR4 found in endothelial cells leading to cell death of the latter cell type. To conclude, TRAIL and TRAILR, being the TNF superfamily members 10, are promising inducers of cell death of both JEV-infected microglia and endothelial cells in receptor independent infection condition.

### Investigation of programmed cell death pathways towards JEV-treated microglia and human microvascular endothelial cells

**Figure 6:**
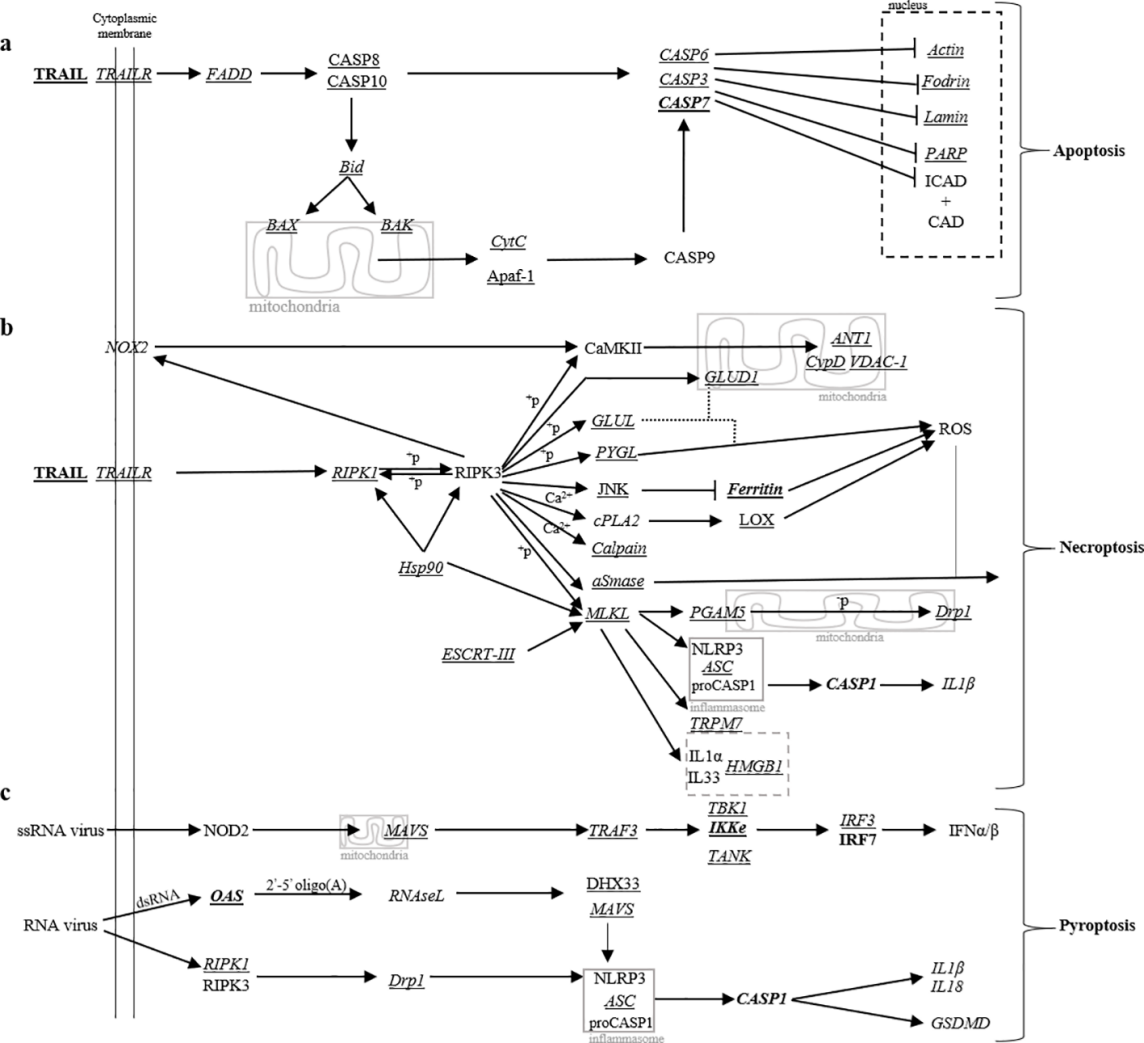
Schematic representation of programmed cell death pathways. **(a)** Apoptotis, **(b)** necroptosis and **(c)** pyroptosis pathways related to TRAIL and RNA viruses triggering are adapted from corresponding human KEGG pathways on GenomeNet (www.genome.jp; hsa04210, hsa04217 and hsa04621). Data summarize transcriptomic analysis as previously done in **Figure 4**, **Figure 5**; **Supplementary Figure 3**. Genes of corresponding protein names detailed in **Tables 1** and **2** and expressed and/or modulated, are highlighted: underlined are genes expressed by human microvascular endothelial cell; in italic are genes expressed mock-treated microglia; and in bold are genes regulated by JEV in human microglia.

Programmed cell death can happen through various pathways including apoptosis, necroptosis and/or pyroptosis with or without implication of the inflammasome for the latter two. The various pathways were investigated via the activity of TRAIL/TRAILR and in the context of RNA and ssRNA viruses as JEV is. Together with **Table 1**, **Table 2** relates gene names with corresponding protein names as used in **Figure 6** and further description.

**Table 2:** List of programmed cell death pathways associated genes including apoptosis, necroptosis, pyroptosis and inflammasome.

Process	Gene name	Protein Name	Ensembl gene ID
Apoptosis	*FADD*	Fas Associated via Death Domain (FADD)	ENSG00000168040
	*BID*	BH3 Interactin Domain Death Antaonist (Bid)	ENSG00000015475
	*BAX*	BCL2 Associated X Apoptosis Reulator (BAX)	ENSG00000087088
	*BAK1*	BCL2 homologous Antaonist killer (BAK)	ENSG00000030110
	*CYCS*	Cytochrome c (CytC)	ENSG00000172115
	*APAF1*	Apoptotic Peptidase Activatin Factor 1 (Apaf-1)	ENSG00000120868
	*ACTB*	Actin β	ENSG00000075624
	*SPTAN1*	Spectrin α (Fodrin)	ENSG00000197694
	*LMNA*	Lamin A/C	ENSG00000160789
	*PARP1*	Poly(ADP-Ribose) Polymerase 1 (PARP)	ENSG00000143799
	*DFFB*	Caspase-activated Dnase (CAD)	ENSG00000169598
Necroptosis	*RIPK1*	Receptor Interacting Serine/Threonine Kinase 1 (RIPK1)	ENSG00000137275
	*RIPK3*	RIPK3	ENSG00000129465
	*HSP90AA1*	Heat Shock Proteine (Hsp) 90 Alpha Class A Member 1	ENSG00000080824
	*HSP90AA2*	Hsp90 Alpha Class A Member 2, pseudogene	ENSG00000224411
	*HSP90AB1*	Hsp90 Alpha Class B Member 1	ENSG00000096384
	*HSP90B1*	Hsp90 Beta Member 1	ENSG00000166598
	*TRAP1*	TNF receptor Associated Protein 1	ENSG00000126602
	*MLKL*	Mixed Lineage Kinase Domain Like Pseudokinase (MLKL)	ENSG00000168404
	*CYBB*	NADPH oxidas 2 (NOD2)	ENSG00000165168
	*CAMK2A*	Calcium/Calmodulin Dependent Protein Kinase II Alpha (CaMKII)	ENSG00000070808
	*GLUD1*	Glutamate Dehydrogenase 1 (GLUD1)	ENSG00000148672
	*GLUL*	Glutamate-Ammonia Ligase (GLUL)	ENSG00000135821
	*PYGL*	Glycogen Phosphorylase L (PYGL)	ENSG00000100504
	*MAPK8*	Mitogen-activated protein kinase 8 (JNK)	ENSG00000107643
	*SMPD1*	Acid Sphingomyelinase (aSmase)	ENSG00000166311
	*SLC25A4*	ADP/ATP translocase 1 (ANT1)	ENSG00000151729
	*PPID*	cyclophilin D (CypD)	ENSG00000171497
	*VDAC1*	Voltage-dependent anion-selective channel 1 (VDAC-1)	ENSG00000213585
	*FTH1*	Ferritin heavy chain	ENSG00000167996
	*PLA2G4A*	Cytosolic phospholipase A2 (cPLA2)	ENSG00000116711
	*CAPN1*	Calpain-1 catalytic subunit (Calpain)	ENSG00000014216
	*LOX*	Lysyl oxidase (+D36:D48LOX)	ENSG00000113083
	*TRPM7*	Transient receptor potential cation channel, subfamily M, member 7 (TRPM7)	ENSG00000092439
	*CHMP2A*	Charged multivesicular body protein 2a (CHMP2A/endosomal sorting complexes required for transport member (ESCRT-III))	ENSG00000130724
	*CHMP2B*	CHMP2B/ESCRT-III	ENSG00000083937
	*CHMP3*	CHMP3/ESCRT-III	ENSG00000115561
	*CHMP4A*	CHMP4A/ESCRT-III	ENSG00000254505
	*CHMP4B*	CHMP4B/ESCRT-III	ENSG00000101421
	*CHMP4C*	CHMP4C/ESCRT-III	ENSG00000164695
	*CHMP6*	CHMP6/ESCRT-III	ENSG00000176108
	*PGAM5*	Mitochondrial Serine/threonine-protein phosphatase (PGAM5)	ENSG00000247077
	*DNM1L*	Dynamin-related protein 1 (Drp1)	ENSG00000087470
	*IL33*	IL33	ENSG00000137033
	*HMGB1*	high mobility group box 1 (HMGB1)	ENSG00000189403
	*IL1A*	IL1α	ENSG00000115008
	*ROS1*	Reactive oxygen species (ROS)	ENSG00000047936
Pyroptosis	*OAS1*	2’-5’-oligoadenylate synthetase 1 (OAS)	ENSG00000089127
	*RNASEL*	RNase L	ENSG00000135828
	*DHX33*	DEAH-Box Helicase 33 (DHX33)	ENSG00000005100
	*MAVS*	Mitochondrial antiviral-signaling protein (MAVS)	ENSG00000088888
	*NOD2*	Nucleotide-binding oligomerization domain-containing protein 2 (NOD2)	ENSG00000167207
	*TRAF3*	TNF receptor-associated factor (TRAF3)	ENSG00000131323
	*TANK*	TRAF family member-associated NF-kappa-B activator (TANK)	ENSG00000136560
	*TBK1*	TANK-binding kinase 1 (TBK1)	ENSG00000183735
	*IKBKE*	Inhibitor of nuclear factor kappa-B kinase subunit epsilon (IKKe)	ENSG00000263528
	*IRF3*	Interferon regulatory factor 3 (IRF3)	ENSG00000126456
	*IRF7*	IRF7	ENSG00000185507
	*IFNA1*	Interferon alpha 1 (IFNα)	ENSG00000197919
	*IFNA2*	Interferon alpha 2 (IFNα)	ENSG00000188379
	*IFNA4*	Interferon alpha 4 (IFNα)	ENSG00000236637
	*IFNA5*	Interferon alpha 5 (IFNα)	ENSG00000147873
	*IFNA6*	Interferon alpha 6 (IFNα)	ENSG00000120235
	*IFNA7*	Interferon alpha 7 (IFNα)	ENSG00000214042
	*IFNA8*	Interferon alpha 8 (IFNα)	ENSG00000120242
	*IFNA10*	Interferon alpha 10 (IFNα)	ENSG00000186803
	*IFNA13*	Interferon alpha 13 (IFNα)	ENSG00000233816
	*IFNA14*	Interferon alpha 14 (IFNα)	ENSG00000228083
	*IFNA16*	Interferon alpha 16 (IFNα)	ENSG00000147885
	*IFNA17*	Interferon alpha 17 (IFNα)	ENSG00000234829
	*IFNA21*	Interferon alpha 21 (IFNα)	ENSG00000137080
	*IFNB1*	Interferon beta (IFNβ)	ENSG00000171855
Inflammasome	*NLRP3*	NOD-like receptor family, pyrin domain containing 3 (NLRP3)	ENSG00000162711
	*PYCARD*	Apoptosis-associated speck-like protein containing a CARD (ASC)	ENSG00000103490
	*IL1B*	Interleukin-1 beta (IL1β)	ENSG00000125538
	*IL18*	IL18	ENSG00000150782
	*GSDMD*	Gasdermin D (GSDMD)	ENSG00000104518

The list details processes, gene name with corresponding protein name and linked human Ensembl gene identification (ENSG) in GRCh38 reference bank.

In the context of microglia, mock-treated human microglia constitutively expressed mRNA for the apoptosis-related genes *FADD*, *BID*, *BAX*, *BAK1*, *CYCS*, *ACTB*, *SPATN1*, *LMNA* and *PAPR1*. However, none were regulated by JEV (**Supplementary Figure 3a**). For necroptosis, mock-treated human microglia constitutively expressed mRNA for *RIPK1*, *HSP90AA1*, *HSP90AB1*, *TRAP1*, *MLKL*, *CYBB*, *GLUD1*, *GLUL*, *PYGL*, *SMPD1*, *PPID*, *VDAC*, *FTH1*, *PLA2G4A*, *CAPN1*, *TRPM7*, *CHMP2B*, *CHMP3*, *CHMP6*, *PGAM5* and *DNML1*. Among necroptosis-related genes, JEV significantly upregulated *FTH1* (**Supplementary Figure 3b**). For pyroptosis, mock-treated human microglia constitutively expressed mRNA for *OAS1*, *RNASEL*, *MAVS*, *TRAF3*, *TANK*, *TNK1*, *IKBKE* and *IRF3*. Among pyroptosis-related genes, JEV significantly upregulated *OAS1*, *IKBKE* and *IRF7* (**Supplementary Figure 3c**). In the context of inflammasome, mock-treated human microglia constitutively expressed mRNA for *PYCARD*, *IL1B*, *IL18* and *GSDMD* but none were affected by JEV (**Supplementary Figure 3d**). In parallel, endothelial cells demonstrated consequent RNA expression for various genes related to apoptosis, necroptotosis and pyroptosis (**Supplementary Figure 3**).

In order to investigate possible implication of programmed cell death pathways, **Figure 6** recapitulates the various intracellular actors being found in microglia and endothelial cells. On one hand, microglia could undergo apoptosis since genes for all players in a CASP6, CASP3 and CASP7-dependent pathways and further modifications in intranuclear actors were found. As previously mentioned, although not significant *CASP8* and *CASP10* were increase by JEV. An exception was found with no expression for Caspase-activated DNnase (CAD) (**Figure 6a**). Also, microglia could follow necroptopsis in a MLKL/PGAM5/Drp1 and MLKL/TRPM7 dependent manners. Nevertheless, Ferritin was the most affected by JEV (**Figure 6b**). Otherwise, ssRNA may induce pyroptosis in an IRF7-dependent pathway and IFNα/β (**Figure 6c**). But, no IFNα/β were detected in supernatants of JEV-infected microglia (data not shown). Finally, RNA virus such as JEV could activate the inflammasome in a MLKL-dependent manner in necroptosis and in an OAS/IL1β-dependent manner in pyroptosis (**Figures 6b, c**). Nevertheless, no IL1β were detected in supernatants of JEV-infected microglia (data not shown). Although previous work demonstrated that JEV-infected microglia did not stain for Annexin-V (Lannes et al., 2017b), JEV may initiate apoptosis, necroptosis and pyroptosis in microglia. In parallel, endothelial cell were expressive for actors related to apoptosis, necroptosis and pyroptosis but without implication of the inflammasome (**Figure 6**). Nevertheless, more investigations are required.

## Discussion

JEV neuroinvasion and neuroinflammation are associated with severe JE outcome. The disruption of the BBB is a later clinical manifestation of JEV infection (Li et al., 2015). Microglia are highly reactive immune cells of the CNS clearing neuro-invasive pathogens but activated microglia can become detrimental and contribute to pathogenesis, as uncontrolled inflammatory responses in JE (Lannes et al., 2017a). Our data prove the potency of endothelial cells to rescue infectious virus from JEV-pulsed microglia. Importantly, the present study exposes yet unknown cytotoxic functions of JEV-associated microglia, but not cell-free JEV, toward endothelial cells forming the BBB. We further reveal that the TNF superfamily members 10 interactions, namely TRAIL/TRAILR, may be a key player in endothelial cell death and may represent a potential therapeutic target.

JEV neuroinvasion represents a crucial step in JEV pathogenesis. Possible neuroinvasion mechanisms include transcellular infection of endothelial cells, paracellular infection, transmigration of infected peripheral immune cells and/or amplification of BBB leakiness (Hsieh and St John, 2020). Our data show that endothelial cells viability is intact in receptor-dependent infection by cell-free JEV, supporting the fact that neuroinvasion precedes neuroinflammation and BBB disruption (Li et al., 2015). Actually, JEV may cross the BBB without its disruption (Khou et al., 2021). Transcellular transport of JEV within endothelial cells occurring via transcytosis or infection and resulting in the release of JEV particles in the CNS (Hsieh and St John, 2020) may be a critical mechanism for JEV propagation into the CNS. In that respect, our data demonstrate the productive infection of human microvascular endothelial cells. However, endothelial cells model of the present study may not form a robust BBB model due to too short incubation period before infection (Khou et al., 2021) and the absence of other critical cell types (Gastfriend et al., 2018).

Within the CNS, JEV interacts with many cell types including microglia. JEV induces apoptosis in mouse microglial cell lines and in microglia of macaques after intranasal infection (Myint et al., 2014). In the current study, change in pro-apoptotic gene expression such executioner caspase-7 and inflammatory caspase-1 occurred as early as 24h exposure. Initiator caspase-8 and executioner caspase-3 are found in neurons and perivascular infiltrates as well as initiator caspase-9 in microglia and astrocytes (Myint et al., 2014). No detectable apoptosis is found in JEV-infected human monocyte-derived microglia in early phase (Lannes et al., 2017b), but later phase of infection may display apoptotic/pyroptotic microglia via caspase pathways. Nonetheless, microglia can survive JEV infection (Thongtan et al., 2010; Lannes et al., 2017b).

Microglia highly contributes to the pathogenesis in JE by supporting an uncontrolled neuroinflammation. The detrimental effect of neuroinflammation is the death of neurons. Although JEV is a neurotropic virus killing neuronal cells, neuroinflammation deriving from microglia also contributes to neuronal cell death (Lannes et al., 2017c). Another important feature of JEV-infected microglia is its potential role to serve as Trojan horse in order to infect other cell types. Indeed, JEV uses microglia to transmit virus content to a target cell resulting in virus rescue (Lannes et al., 2019). Human endothelial cells support JEV propagation (Agrawal et al., 2013; Khou et al., 2021) and may be a suitable target cell type in JE pathogenesis.

Although the integrity of the BBB remains intact during the neuroinvasion process by JEV (Khou et al., 2021), JEV ultimately disrupts the BBB leading to its increased permeability (Li et al., 2015). The breakdown of the BBB displays various characteristics including loss of tight junctions and endothelium degeneration (Hussain et al., 2021). Loss of expression of tight junctions such as Occludin, Claudin-5 and ZO-1 correlates with the severity of JE (Li et al., 2015). Our data demonstrate that JEV is cytotoxic to endothelial cells in a receptor-independent manner via JEV-associated microglia. This supports the chronology of neuroinvasion preceding the BBB disruption (Li et al., 2015) for which JEV-associated microglia may contribute by affecting the viability of endothelial cells of the BBB. Although no apoptosis was detected in receptor-dependent infection at low viral load, cell death of endothelial cells may occur at later stage of infection with stronger modulation of pro-apoptotic proteins at low viral load (Al-Obaidi et al., 2017). Nevertheless, the present model lack interactions with other cell types such as astrocytes and pericytes. Indeed, both cell types react to JEV by producing various cytokines which may affect BBB integrity and/or microglial functions (Chen et al., 2014; Mohapatra et al., 2023).

A leading cause of the disruption of the BBB is neuroinflammation, a hallmark of JE in humans. The sources of neuroinflammatory factors are multiple, including microglia, astrocytes and pericytes (Chen et al., 2014; Lannes et al., 2017b; Wang et al., 2018). Inflammatory soluble factors, such as TNFSF2/TNF-α, damage the integrity of the BBB (Wang et al., 2018). CXCL10 promotes the production of TNF-α by astrocytes (Wang et al., 2018). Despite the production of CXCL-10 by JEV-treated microglia (Lannes et al., 2017b) and the increase of TNF-α gene expression in the present study, supernatant and its content were inefficient in inducing apoptosis of endothelial cells. Nevertheless, the effect of TNF-α may be more significant on other components of the BBB disruption such as loss of tight junctions’ function and/or at unreached sensitivity in the present experimental settings. The present study identified the TNFSF superfamily members 10, known as TRAIL/TRAILR, as a potential therapeutic target in JE. TRAIL is a key player in the apoptosis pathway if interacting with at least one of its agonistic receptors TRAILR1 and TRAILR2. However, a tight regulation exists through the antagonistic receptors TRAILR3 and TRAILR4 (Alves et al., 2021). Although TRAIL could mediate apoptosis in endothelial cells (O’Brien et al., 2007), TRAIL may be protective against endothelial dysfunction *in vivo* (Manuneedhi Cholan et al., 2018).

In the present study, cell death may be initiated by TRAIL/TRAILR and/or RNA viral molecules. Via TRAIL, a possible apoptosis pathway is through FADD and/or BAX with further Caspase activity, especially Caspase 7. Indeed, JEV causes Caspase 3/7-mediated apoptosis via the BAX-mediated release of Cytochrome C from mitochondria to the cytoplasm (Ashraf et al., 2021; Yang et al., 2024). However, JEV activates Caspase 8 in a FADD-independent manner (Tsao et al., 2008). TRAIL may also induce necroptosis via RIPK1/3 and MLKL. In JEV infection, MLKL-dependent necroptosis has been shown in mice (Bian et al., 2017). And MLKL-dependent pathway requires RPIK1/3 (Yuan et al., 2019). In addition, TRAIL may initiate necroptosis via Ferritin activity in endothelial cells and intracellular accumulation of iron leads to inflammatory and oxidative status in JEV infection *in vivo* (Singh et al., 2024). Pyroptosis by sensing RNA material from JEV may happen through IRF7 and/or OAS. Actually, *OAS1* contribute to neuroinflammation and antiviral state in microglia (Mishra et al., 2022). Supporting our data, apoptosis, necroptosis and pyroptosis were suggested in a JEV infection model of peritoneal macrophages (Wang et al., 2020). However, the exact mechanism of TRAIL-related cell death requires more investigations to be elucidated in the context of JE.

Microglia functions represent critical therapeutic targets in fighting JE. Microglia demonstrate key contribution in the pathogenesis and severity of JE through the involvement in neuroinflammation, the supportive role in JEV propagation in a receptor-independent manner, the potential of being a Trojan Horse and the cytotoxicity towards endothelial cells. Microglia derived-neuroinflammation can be specifically targeted by anti-inflammatory molecules resulting in various efficiencies (Lannes et al., 2017c). Intercellular virus transmission by microglia is partially dependent to the CX_3_CR1/CX_3_CL1 axis of which targeting results in strong inhibition of JEV rescue (Lannes et al., 2019). Nevertheless, the present study highlights a cytotoxic effect of the CX_3_CR1 antagonist used here, probably limiting its effect. Still, more work is necessary to better define the advantages and limits of such therapeutic candidate. Aiming the Trojan horse function of microglia must require specific antiviral therapeutics, possibly targeting essential elements for replication of ssRNA viruses, including JEV. The present study exposes the cytotoxicity of JEV-associated microglia towards endothelial cells in a receptor-independent infection manner. This last process might be targeted by therapeutic approach directed to TRAIL activity.

## Data Availability

The data of RNA sequencing presented in the study are deposited in the Zenodo.org repository, accession number: https://doi.org/10.5281/zenodo.15065414. Other datasets are available upon request.

## Supplementary material

Supplementary Figure 1 JEV-pulsed microglia supernatant dependent cytotoxicity on endothelial cells. Scatter dot plot representing the frequencies of Annexin-V^+^ single microvascular endothelial cells cultured in supernatants of mock and JEV-treated microglia, at an MOI of 10 TCID_50_/cell for 6 days. Data are of independent experiments with each condition performed in triplicate, the solid line is the mean value and the dashed line is the baseline (y=0%). Each colour represents a blood donor (#4). Asterisks show significant differences using the unpaired t-test (*: p<0.05; **: p<0.01; ***: p<0.001; ****: p<0.0001).

Supplementary Figure 2 Identification of human microglial cells and associated cell death in receptor-independent infection culture system. **(a)** Merged dot plot FSC/SSC representation of human microglia (red) and human endothelial cells (blue) on flow cytometry after single cell identification. Gates represent selected microglia and endothelial cells based on their SSC-A/FSC-A profile (left panel). Subsequent histogram representations for the expression for CD11b, CX_3_CR1 and CD31 on microglia (filled red) and microvascular endothelial cells (filled blue) including the isotype control (open black) (right panels). **(b)** Representative gating strategy for flow cytometry analysis of Annexin-V staining in receptor-independent infection of microvascular endothelial cells with mock- and JEV-associated microglia, with a MOI of 10 TCID_50_/cell for 6 days. Selected microglia cells based on FSC/SSC profile after debris exclusion (left panel) and single cells selection (middle panel) are shown in pseudo-plot representation. Subsequent histogram plot shows frequencies of Annexin-V^+^ microglia cells (right panel). **(c)** Scatter dot plot representing the frequencies of Annexin-V^+^ single microglia cells in a receptor-independent infection, as described in **(b)**. **(d)** Scatter dot plot representing the frequencies of Annexin-V^+^ single microvascular endothelial cells in a receptor-independent infection of microvascular endothelial cells mock and JEV-associated microglia, with a MOI of 10 TCID_50_/cell for 6 days, in presence of DMSO and indicated concentration of CX_3_CR1 antagonist, as gated in **(b)**. Data are of independent experiments with each condition performed in triplicate, the solid line is the mean value and the dashed line is the baseline (y=0%). **(b)** Each colour represents a blood donor (#5) and **(d)** each symbol is a replicate from 3 blood donors. Asterisks show significant differences using the unpaired t-test (*: p<0.05; **: p<0.01; ***: p<0.001; ****: p<0.0001).

Supplementary Figure 3 Transcriptomic analysis of programmed cell death genes in human microglia and human microvascular endothelial cells. Scatter dot plot representing gene count in FPKM of human **(a)** apoptosis, **(b)** necroptosis, **(c)** pyroptosis and **(d)** inflammasome genes after transcriptomic analysis in mock- and JEV-treated microglia and in human microvascular endothelial cells at steady state. Data are of 3 independent experiments the dashed line is the baseline (= 5 FPKM). For microglia model, each symbol represents a blood donor in which cells have treated with mock or JEV. For human microvascular endothelial cells, each symbol represents a batch of the cell culture. Statistics are calculated with the 2-way ANOVA test (*: p<0.05; **: p<0.01; ***: p<0.001). **(c)** Summary table of proteins interactions between TNF superfamily ligands with TNF superfamily receptors. Genes of corresponding protein names detailed in **Table 1** and expressed and/or modulated in **Figure 5a, b**, are highlighted: underlined are genes expressed by human microvascular endothelial cell; in italic are genes expressed mock-treated microglia; and in bold are genes regulated by JEV in human microglia.

## References

[B1] Agrawal T. Sharvani V. Nair D. Medigeshi G. R. (2013). Japanese encephalitis virus disrupts cell-cell junctions and affects the epithelial permeability barrier functions. PloS One 8, e69465. 23894488 10.1371/journal.pone.0069465 PMC3722119

[B2] Al-Obaidi M. M. J. Bahadoran A. Har L. S. Mui W. S. Rajarajeswaran J. Zandi K.. (2017). Japanese encephalitis virus disrupts blood-brain barrier and modulates apoptosis proteins in THBMEC cells. Virus Res. 233, 17–28. 28279803 10.1016/j.virusres.2017.02.012

[B3] Alves L. C. Corazza N. Micheau O. Krebs P. (2021). The multifaceted role of TRAIL signaling in cancer and immunity. FEBS J. 288, 5530–5554. 33215853 10.1111/febs.15637

[B4] Ashraf U. Ding Z. Deng S. Ye J. Cao S. Chen Z. (2021). Pathogenicity and virulence of Japanese encephalitis virus: Neuroinflammation and neuronal cell damage. Virulence. 12, 968–980. 33724154 10.1080/21505594.2021.1899674 PMC7971234

[B5] Bian P. Zheng X. Wei L. Ye C. Fan H. Cai Y.. (2017). MLKL mediated necroptosis accelerates JEV-induced neuroinflammation in mice. Front. Microbiol. 8, 303. 28293227 10.3389/fmicb.2017.00303 PMC5328978

[B6] Chen C. J. Ou Y. C. Li J. R. Chang C. Y. Pan H. C. Lai C. Y.. (2014). Infection of pericytes *in vitro* by Japanese encephalitis virus disrupts the integrity of the endothelial barrier. J. virology. 88, 1150–1161. 24198423 10.1128/JVI.02738-13 PMC3911661

[B7] Etemad S. Zamin R. M. Ruitenberg M. J. Filgueira L. (2012). A novel *in vitro* human microglia model: characterization of human monocyte-derived microglia. J. Neurosci. Methods 209, 79–89. 22659341 10.1016/j.jneumeth.2012.05.025

[B8] Filgueira L. Lannes N. (2019). Review of Emerging Japanese Encephalitis Virus: New Aspects and Concepts about Entry into the Brain and Inter-Cellular Spreading. Pathogens. 8 (3), 111. doi: 10.3390/pathogens8030111 31357540 PMC6789543

[B9] Gastfriend B. D. Palecek S. P. Shusta E. V. (2018). Modeling the blood-brain barrier: Beyond the endothelial cells. Curr. Opin. BioMed. Eng. 5, 6–12. 29915815 10.1016/j.cobme.2017.11.002 PMC6003712

[B10] Ginhoux F. Lim S. Hoeffel G. Low D. Huber T. (2013). Origin and differentiation of microglia. Front. Cell. Neurosci. 7, 45. 23616747 10.3389/fncel.2013.00045 PMC3627983

[B11] Gossner C. M. Dhollander S. Presser L. D. Briet O. Bakonyi T. Schaffner F.. (2024). Potential for emergence of Japanese encephalitis in the European Union. Zoonoses Public Health 71, 274–280. 38110840 10.1111/zph.13103

[B12] Hsieh J. T. St John A. L. (2020). Japanese encephalitis virus and its mechanisms of neuroinvasion. PloS pathogens. 16, e1008260. 32240272 10.1371/journal.ppat.1008260 PMC7117652

[B13] Hulshof S. van Haastert E. S. Kuipers H. F. van den Elsen P. J. De Groot C. J. van der Valk P.. (2003). CX3CL1 and CX3CR1 expression in human brain tissue: noninflammatory control versus multiple sclerosis. J. neuropathology Exp. neurology. 62, 899–907. 10.1093/jnen/62.9.899 14533779

[B14] Hussain B. Fang C. Chang J. (2021). Blood-brain barrier breakdown: an emerging biomarker of cognitive impairment in normal aging and dementia. Front. Neurosci. 15, 688090. 34489623 10.3389/fnins.2021.688090 PMC8418300

[B15] Khou C. Diaz-Salinas M. A. da Costa A. Prehaud C. Jeannin P. Afonso P. V.. (2021). Comparative analysis of neuroinvasion by Japanese encephalitis virulent and vaccine viral strains in an *in vitro* model of human blood-brain barrier. PloS One 16, e0252595. 34086776 10.1371/journal.pone.0252595 PMC8177624

[B16] Kimura-Kuroda J. Ichikawa M. Ogata A. Nagashima K. Yasui K. (1993). Specific tropism of Japanese encephalitis virus for developing neurons in primary rat brain culture. Arch. Virol. 130, 477–484. 8517798 10.1007/BF01309676 PMC7086854

[B17] Lannes N. Eppler E. Etemad S. Yotovski P. Filgueira L. (2017a). Microglia at center stage: a comprehensive review about the versatile and unique residential macrophages of the central nervous system. Oncotarget. 8, 114393–114413. 29371994 10.18632/oncotarget.23106 PMC5768411

[B18] Lannes N. Garcia-Nicolas O. Demoulins T. Summerfield A. Filgueira L. (2019). CX3CR1-CX3CL1-dependent cell-to-cell Japanese encephalitis virus transmission by human microglial cells. Sci. Rep. 9, 4833. 30886214 10.1038/s41598-019-41302-1 PMC6423114

[B19] Lannes N. Neuhaus V. Scolari B. Kharoubi-Hess S. Walch M. Summerfield A.. (2017b). Interactions of human microglia cells with Japanese encephalitis virus. Virol. J. 14, 8. 28088249 10.1186/s12985-016-0675-3 PMC5237516

[B20] Lannes N. Summerfield A. Filgueira L. (2017c). Regulation of inflammation in Japanese encephalitis. J. neuroinflammation. 14, 158. 28807053 10.1186/s12974-017-0931-5 PMC5557552

[B21] Li F. Wang Y. Yu L. Cao S. Wang K. Yuan J.. (2015). Viral infection of the central nervous system and neuroinflammation precede blood-brain barrier disruption during Japanese encephalitis virus infection. J. virology. 89, 5602–5614. 25762733 10.1128/JVI.00143-15 PMC4442524

[B22] Manuneedhi Cholan P. Cartland S. P. Dang L. Rayner B. S. Patel S. Thomas S. R.. (2018). TRAIL protects against endothelial dysfunction *in vivo* and inhibits angiotensin-II-induced oxidative stress in vascular endothelial cells *in vitro* . Free Radical Biol. Med. 126, 341–349. 30165101 10.1016/j.freeradbiomed.2018.08.031

[B23] Mishra R. Kumawat K. L. Basu A. Banerjea A. C. (2022). Japanese Encephalitis Virus infection increases USP42 to stabilize TRIM21 and OAS1 for neuroinflammatory and anti-viral response in human microglia. Virology. 573, 131–140. 35779335 10.1016/j.virol.2022.06.012

[B24] Misra U. K. Kalita J. (2010). Overview: Japanese encephalitis. Prog. neurobiology. 91, 108–120. 10.1016/j.pneurobio.2010.01.008 20132860

[B25] Mohapatra S. Chakraborty T. Basu A. (2023). Japanese Encephalitis virus infection in astrocytes modulate microglial function: Correlation with inflammation and oxidative stress. Cytokine. 170, 156328. 37567102 10.1016/j.cyto.2023.156328

[B26] Monath T. P. (2023). Japanese encephalitis: risk of emergence in the United States and the resulting impact. Viruses 16 (1), 54. doi: 10.3390/v16010054 38257754 PMC10820346

[B27] Myint K. S. Kipar A. Jarman R. G. Gibbons R. V. Perng G. C. Flanagan B.. (2014). Neuropathogenesis of Japanese encephalitis in a primate model. PloS Negl. Trop. diseases. 8, e2980. 10.1371/journal.pntd.0002980 PMC4125110 25102067

[B28] O’Brien L. A. Richardson M. A. Mehrbod S. F. Berg D. T. Gerlitz B. Gupta A.. (2007). Activated protein C decreases tumor necrosis factor related apoptosis-inducing ligand by an EPCR- independent mechanism involving Egr-1/Erk-1/2 activation. Arteriosclerosis thrombosis Vasc. Biol. 27, 2634–2641. 10.1161/ATVBAHA.107.153734 17932312

[B29] Ricklin M. E. García-Nicolás O. Brechbühl D. Python S. Zumkehr B. Nougairede A.. (2016). Vector-free transmission and persistence of Japanese encephalitis virus in pigs. Nat Commun 7, 10832. doi: 10.1038/ncomms10832 26902924 PMC4766424

[B30] Sheikh B. N. Bondareva O. Guhathakurta S. Tsang T. H. Sikora K. Aizarani N.. (2019). Systematic identification of cell-cell communication networks in the developing brain. iScience. 21, 273–287. 31677479 10.1016/j.isci.2019.10.026 PMC6838536

[B31] Shwetank Date O. S. Kim K. S. Manjunath R. (2013). Infection of human endothelial cells by Japanese encephalitis virus: increased expression and release of soluble HLA-E. PloS One 8, e79197. 24236107 10.1371/journal.pone.0079197 PMC3827286

[B32] Singh G. Singh A. Mishra S. Singh D. Kumar A. (2024). Intracellular iron accumulation induces inflammatory and oxidative status of the host after Japanese encephalitis viral infection. Mol. neurobiology. 61, 175–187. 10.1007/s12035-023-03538-x 37594653

[B33] Solomon T. (2004). Flavivirus encephalitis. New Engl. J. Med. 351, 370–378. 15269317 10.1056/NEJMra030476

[B34] Srivastava K. S. Jeswani V. Pal N. Bohra B. Vishwakarma V. Bapat A. A.. (2023). Japanese encephalitis virus: an update on the potential antivirals and vaccines. Vaccines (Basel) 11 (4), 472. doi: 10.3390/vaccines11040742 37112654 PMC10146181

[B35] Thongtan T. Cheepsunthorn P. Chaiworakul V. Rattanarungsan C. Wikan N. Smith D. R. (2010). Highly permissive infection of microglial cells by Japanese encephalitis virus: a possible role as a viral reservoir. Microbes infection/Institut Pasteur. 12, 37–45. 10.1016/j.micinf.2009.09.013 19786116

[B36] Thongtan T. Thepparit C. Smith D. R. (2012). The involvement of microglial cells in Japanese encephalitis infections. Clin. Dev. Immunol. 2012, 890586. 22919405 10.1155/2012/890586 PMC3420229

[B37] Tsao C. H. Su H. L. Lin Y. L. Yu H. P. Kuo S. M. Shen C. I.. (2008). Japanese encephalitis virus infection activates caspase-8 and -9 in a FADD-independent and mitochondrion-dependent manner. J. Gen. Virol. 89, 1930–1941. 18632964 10.1099/vir.0.2008/000182-0

[B38] Wang H. Liang G. (2015). Epidemiology of Japanese encephalitis: past, present, and future prospects. Ther. Clin. Risk management. 11, 435–448. 10.2147/TCRM.S51168 PMC4373597 25848290

[B39] Wang K. Wang H. Lou W. Ma L. Li Y. Zhang N.. (2018). IP-10 promotes blood-brain barrier damage by inducing tumor necrosis factor alpha production in Japanese encephalitis. Front. Immunol. 9, 1148. 29910805 10.3389/fimmu.2018.01148 PMC5992377

[B40] Wang Z. Y. Zhen Z. D. Fan D. Y. Wang P. G. An J. (2020). Transcriptomic analysis suggests the M1 polarization and launch of diverse programmed cell death pathways in Japanese encephalitis virus-infected macrophages. Viruses 12 (12), 356. doi: 10.3390/v12030356 32213866 PMC7150907

[B41] Yang K. Li X. Yang S. Zheng Y. Cao S. Yan Q.. (2024). Japanese encephalitis virus infection induces mitochondrial-mediated apoptosis through the proapoptotic protein BAX. Front. Microbiol. 15, 1485667. 39529669 10.3389/fmicb.2024.1485667 PMC11550975

[B42] Yuan J. Amin P. Ofengeim D. (2019). Necroptosis and RIPK1-mediated neuroinflammation in CNS diseases. Nat. Rev. Neurosci. 20, 19–33. 30467385 10.1038/s41583-018-0093-1 PMC6342007

[B43] Yun S. I. Lee Y. M. (2018). Early events in Japanese encephalitis virus infection: viral entry. Pathogens. 7 (3), 68. doi: 10.3390/pathogens7030068 30104482 PMC6161159

[B44] Zhang C. Zhang Y. Zhuang R. Yang K. Chen L. Jin B.. (2024). Alterations in CX3CL1 levels and its role in viral pathogenesis. Int. J. Mol. Sci. 25(8), 4451. doi: 10.3390/ijms25084451 38674036 PMC11050295

