# Supplementary figures and images for: Japanese encephalitis virus-associated human microglia induce cell death of human microvascular endothelial cells in receptor-independent infection
Source: Front Cell Infect Microbiol. 2025 May 2;15:1580958. doi: 10.3389/fcimb.2025.1580958 (PMC12081440; doi:10.3389/fcimb.2025.1580958)

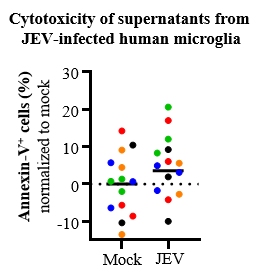

Supplement: Supplementary Figure 1 — JEV-pulsed microglia supernatant dependent cytotoxicity on endothelial cells. Scatter dot plot representing the frequencies of Annexin-V+ single microvascular endothelial cells cultured in supernatants of mock and JEV-treated microglia, at an MOI of 10 TCID50/cell for 6 days. Data are of independent experiments with each condition performed in triplicate, the solid line is the mean value and the dashed line is the baseline (y=0%). Each colour represents a blood donor (#4). Asterisks show significant differences using the unpaired t-test (*: p<0.05; **: p<0.01; ***: p<0.001; ****: p<0.0001). [file Image1.tif]

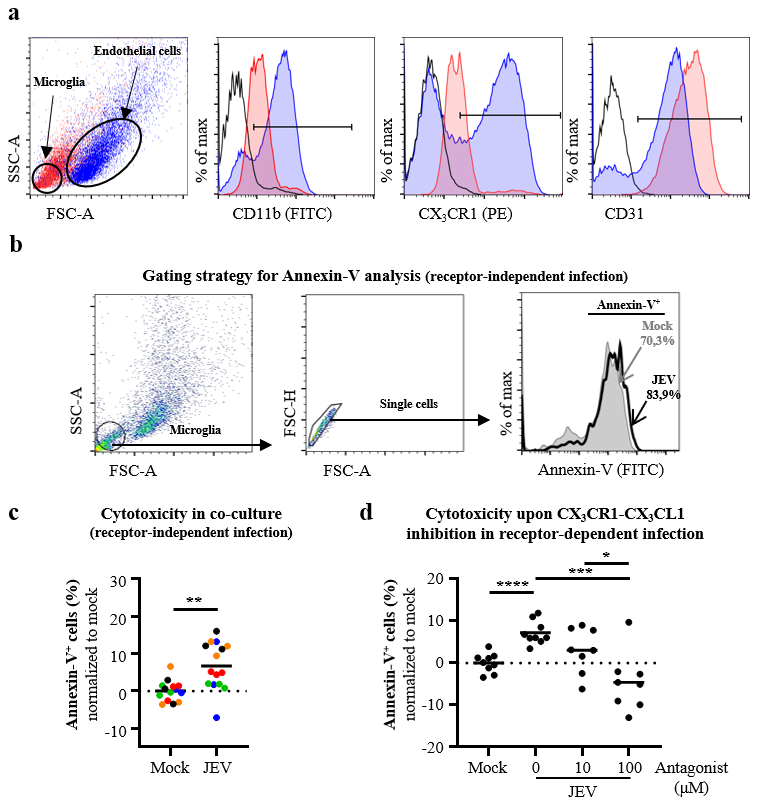

Supplement: Supplementary Figure 2 — Identification of human microglial cells and associated cell death in receptor-independent infection culture system. (a) Merged dot plot FSC/SSC representation of human microglia (red) and human endothelial cells (blue) on flow cytometry after single cell identification. Gates represent selected microglia and endothelial cells based on their SSC-A/FSC-A profile (left panel). Subsequent histogram representations for the expression for CD11b, CX3CR1 and CD31 on microglia (filled red) and microvascular endothelial cells (filled blue) including the isotype control (open black) (right panels). (b) Representative gating strategy for flow cytometry analysis of Annexin-V staining in receptor-independent infection of microvascular endothelial cells with mock- and JEV-associated microglia, with a MOI of 10 TCID50/cell for 6 days. Selected microglia cells based on FSC/SSC profile after debris exclusion (left panel) and single cells selection (middle panel) are shown in pseudo-plot representation. Subsequent histogram plot shows frequencies of Annexin-V+ microglia cells (right panel). (c) Scatter dot plot representing the frequencies of Annexin-V+ single microglia cells in a receptor-independent infection, as described in (b). (d) Scatter dot plot representing the frequencies of Annexin-V+ single microvascular endothelial cells in a receptor-independent infection of microvascular endothelial cells mock and JEV-associated microglia, with a MOI of 10 TCID50/cell for 6 days, in presence of DMSO and indicated concentration of CX3CR1 antagonist, as gated in (b). Data are of independent experiments with each condition performed in triplicate, the solid line is the mean value and the dashed line is the baseline (y=0%). (b) Each colour represents a blood donor (#5) and (d) each symbol is a replicate from 3 blood donors. Asterisks show significant differences using the unpaired t-test (*: p<0.05; **: p<0.01; ***: p<0.001; ****: p<0.0001). [file Image2.tif]

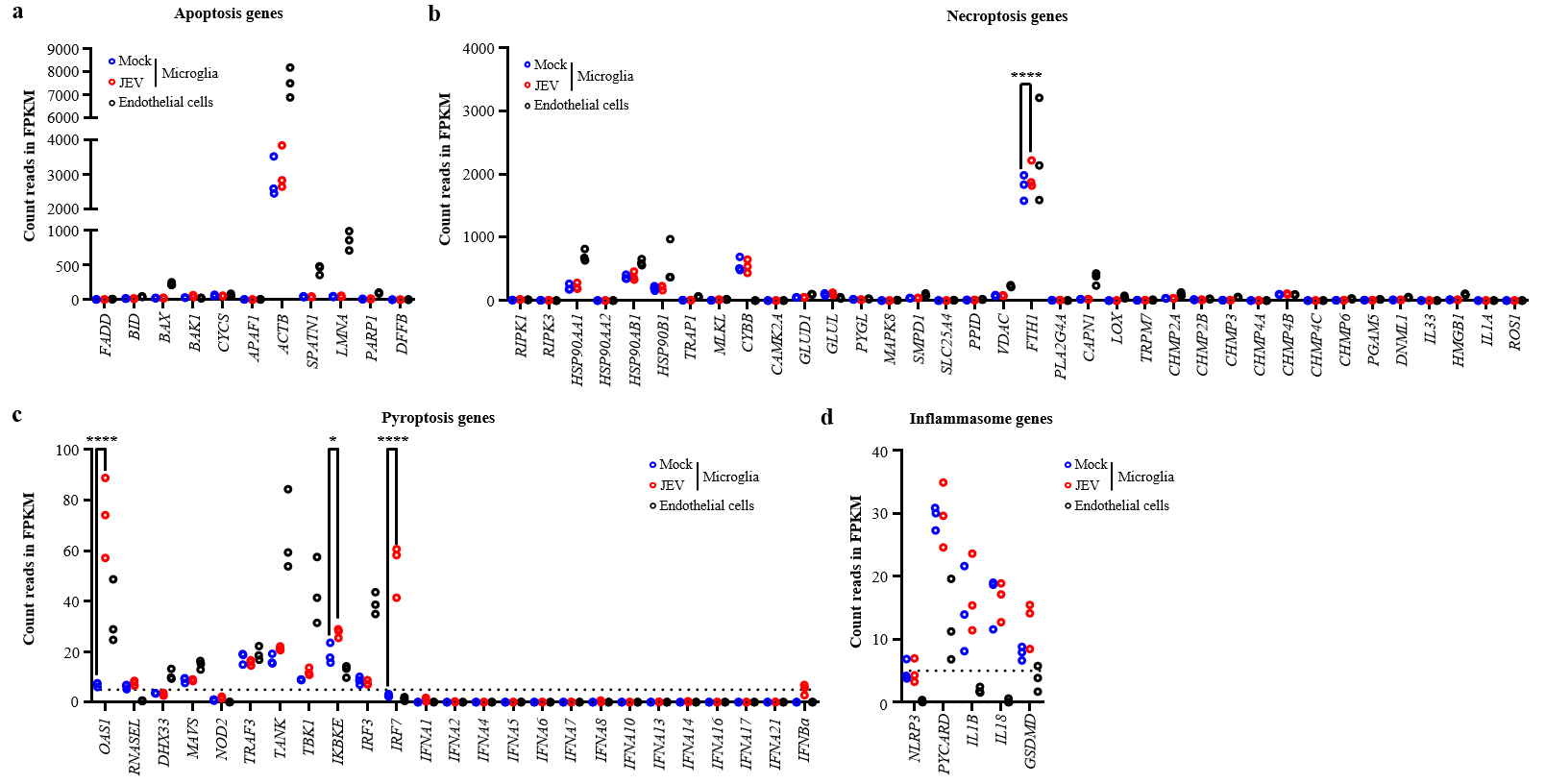

Supplement: Supplementary Figure 3 — Transcriptomic analysis of programmed cell death genes in human microglia and human microvascular endothelial cells. Scatter dot plot representing gene count in FPKM of human (a) apoptosis, (b) necroptosis, (c) pyroptosis and (d) inflammasome genes after transcriptomic analysis in mock- and JEV-treated microglia and in human microvascular endothelial cells at steady state. Data are of 3 independent experiments the dashed line is the baseline (= 5 FPKM). For microglia model, each symbol represents a blood donor in which cells have treated with mock or JEV. For human microvascular endothelial cells, each symbol represents a batch of the cell culture. Statistics are calculated with the 2-way ANOVA test (*: p<0.05; **: p<0.01; ***: p<0.001). (c) Summary table of proteins interactions between TNF superfamily ligands with TNF superfamily receptors. Genes of corresponding protein names detailed in Table 1 and expressed and/or modulated in Figure 5a, b , are highlighted: underlined are genes expressed by human microvascular endothelial cell; in italic are genes expressed mock-treated microglia; and in bold are genes regulated by JEV in human microglia. [file Image3.tif]
